# Precision Grafting-From of Diblock Copolymer Brushes on MXene Nanosheets

**DOI:** 10.1021/acs.chemmater.5c01572

**Published:** 2025-10-13

**Authors:** Jinyoung Choi, Mykhailo Yelipashev, Valeriia Poliukhova, James FitzPatrick, Yury Gogotsi, Zhiqun Lin, Vladimir V. Tsukruk

**Affiliations:** † School of Materials Science and Engineering, 1372 Georgia Institute of Technology, Atlanta, Georgia 30332, United States; ‡ A. J. Drexel Nanomaterials Institute and Department of Materials Science and Engineering, 6527 Drexel University, Philadelphia, Pennsylvania 19104, United States; § Department of Chemical and Biomolecular Engineering, 37580 National University of Singapore, Singapore, 117585 Singapore

## Abstract

2D MXene materials offer outstanding optical, electrical, and mechanical properties, which can be used to produce multifunctional high-performance polymer–matrix composites. Here, we demonstrate the fabrication of robust covalently bonded polymer shells via the implementation of surface-initiated atom transfer radical polymerization (SI-ATRP) on heterogeneous 2D nanosheets. This robust grafting-from methodology was demonstrated through selective esterification of hydroxyl groups on Ti_3_C_2_T*_*x*_* MXene with 2-bromoisobutyryl bromide. This approach enables the synthesis of diblock polymer brushes with sequential hydrophobic and hydrophilic blocks with predetermined, narrowly dispersed molecular weight and a low polydispersity index firmly bonded to the nanosheet surface. The high molecular weight of diblock copolymers was achieved by a precise design of molecular compositions (block ratio). By combining selective chain cleavage and monitoring the evolution of polymer shell morphology, we confirmed that the growth of polymer brushes falls in the near-brush regime with a high grafting density of 0.18–0.25 chains/nm^2^ and shell thickness of 20–50 nm. Compared to common grafting-to and physical adsorption processes, the key benefits of this grafting-from approach lie in combining the 2D MXene nanosheets with the versatility of firmly grafted diblock copolymer functionalities and a core–shell brush architecture where MXene’s inherent structure is protected in harsh chemical environments.

## Graphical abstract

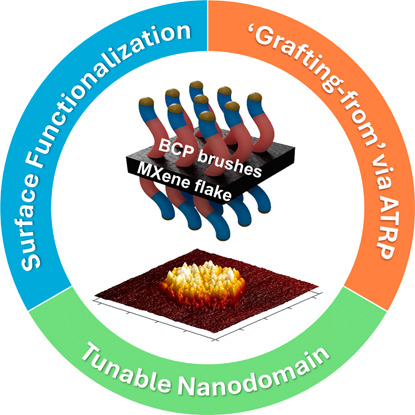

## Introduction

MXenes, a broad class of two-dimensional (2D) carbides or nitrides, are emerging materials for multiple technological domains due to their unique properties.^1–4^ High electrical conductivity, mechanical strength, and rich surface terminations with diverse functionality, in conjunction with their intrinsic 2D morphology, position MXenes as promising functional components of composite nanomaterials for energy storage, wearable and flexible electronics, wireless communication, biofunctional materials, and sensing applications.^5–9^

The defining characteristic of MXenes produced by selective wet chemical etching of layered MAX phase precursors lies in their abundant functional surface terminations (T_*x*_ = −OH, −O, and −F), which can serve as a bridge for precise surface engineering and tailored interface interactions with surroundings.^10,11^ However, the distribution of functional groups on MXene nanosheets depends on etching, synthesis, delamination, and post-treatment processes,^12,13^ including a persistent challenge in MXenes’ propensity to aggregate in nonaqueous environments due to reduced surface charge, which hinders controlled surface functionalization and performance in composite systems. Consequently, the ability to modulate the surface-mediated interactions with the surrounding represents a critical frontier in the tailored development of MXene-based hybrid materials.

Integration of MXenes with diblock copolymers, which are macromolecules consisting of two distinct polymer segments covalently linked together, offers advantages that may enhance MXene performance beyond that achieved by functionalization with small organic molecules^14^ and physically adsorbed shells.^15^ The precise macromolecular structures of diblock copolymers with adjustable chain architectures provide unique capabilities, including precisely controllable chain architectures, stimuli-responsiveness, and the ability to self-assemble into well-defined nanodomains.^16,17^ The system also offers tunable properties such as selective hydrophilicity/hydrophobicity, mechanical reinforcement, responsive behavior, and programmable binding affinities.^18–20^ However, current approaches to fabricating this MXene–polymer heterostructure using diblock copolymers have predominantly relied on simplistic strategies like direct physical mixing and assembly or noncovalent surface modifications.^21,22^ These methods provide limited control over polymer architecture, interfacial interactions, and uneven surface terminations on MXene sheets, resulting in weak bonding, low shell stability, and suboptimal performance under variable external stimuli such as solvent quality or temperature.

Meanwhile, among the approaches for covalently binding polymers to interfaces, atom transfer radical polymerization (ATRP) offers exceptional control over polymer molecular weight, composition, and architecture during grafting of polymer brushes on a functionalized surface.^23,24^ While ATRP has been successfully applied to traditional 2D materials like graphene oxide^25^ and boron nitride^26^ for solubility enhancement, improving thermal conductivity, or increasing toughness, it remains an underexplored area for MXene modification.^27,28^ Despite the maturity of ATRP and surface grafting on 2D nanosheets separately, the integration specifically on MXene surfaces remains a largely understudied area with minimal research precedents. The gap is likely to stem from unique MXene-specific challenges, for example, achieving heterogeneous surface exposure in nonpolar solvent environment and uniform initiator coverage during surface functionalization. Moreover, the inherent complexity of precisely characterizing polymer brushes on such reactive surfaces creates significant barriers to systematic investigation and validation of controlled polymerization on MXenes via grafting-from method. Therefore, advancing a fundamental understanding and strategies for polymer integration and growth dynamics on conductive substrates, like MXene, with interfacial engineering is essential for the future development of adaptive nanocomposite materials. Critical aspects such as controlled grafting densities, chain length tunability, surface morphology, and block sequence manipulation must be considered for material performance. Additionally, the strategic design of effective ATRP initiators that directly correspond to MXene’s irregular surface functional groups should be explored to obtain uniform and controllable polymer grafting.

Herein, we address these critical limitations by developing a comprehensive route comprising a targeted methodology to maximize surface grafting density on the complex surface of MXene nanosheets to covalently graft and analyze well-defined diblock copolymers composed of poly­(4-vinylpyridine) (P4VP), poly­(*tert*-butyl acrylate) (PtBA), and polystyrene (PS) blocks by the grafting-from approach. The most distinguished findings of this work include in-depth confirmation of structural and functional integrity of the system, demonstrating transformative advantages offered by the SI-ATRP, including enhanced interfacial stability through strong covalent bonds instead of weak physical interactions. Among these are control over polymer nanodomain morphology, optimizing charge transport pathways; selective surface functionality through tailored block chemistry that modulates the work function, hydrophilicity, and reactivity without compromising binding to nanosheet surfaces; and improved protection while maintaining intrinsic MXene properties. This approach allows diversely functionalized MXenes to form sophisticated composite structures through tailored interactions with surrounding polymer matrices, including weak interactions, in situ growth, and covalent bonding. They can be used as next-generation functional multicomponent materials with tailored properties for biomedical applications, catalysis, flexible electronics, supercapacitors, batteries, sensors, and more.^29–31^

Unlike most conventional methods that merely encapsulate MXenes, this grafting-from architecture creates an intricate chemical interface that enables control over the properties of the resulting organic–inorganic nanocomposites. This work focuses on the synthetic methodology and characterization of the obtained MXene–polymer nanohybrids, where we investigated the polymerization kinetics and control over polymer architecture on MXene nanosheet surfaces and established a direct correlation between synthesis parameters and structural characteristics on resulting grafted-from polymer layers, including expected linear relationships between chain length and reaction time or temperature, along with controlled low polydispersity and morphologies. Furthermore, the structural and functional advantages of the MXene surface are complemented, as the covalent binding of hydroxyl (−OH) groups on MXene surface to ATRP initiator ensures enhanced interfacial adhesion and stability of the grafted polymer brushes, allowing us to fully exploit MXenes’ inherent properties while incorporating the functionality and versatility of synthetic polymers such as tunable surface protection and surface property control. The results indicate promising approaches for preprogrammed fabrication of robust functional MXene-based organic–inorganic hybrid nanomaterials with stable and adjustable surface properties and molecular level interfacial interactions.

## Results and Discussion

The schematic of the ATRP-based approach to fabricate the MXene–polymer hybrids is depicted in Figure 1, describing step-by-step procedures to functionalize MXene surface and graft first and second polymer blocks. Ti_3_C_2_T_*x*_ MXene nanosheets were prepared through the previously reported method.^32^ The MXene sheet possesses abundant surface terminations, including – OH groups, which are key factors in our approach as they serve as reactive sites for targeted surface functionalization (Figure 1a). Single and double-layer MXene 2D flakes with typical thicknesses and lateral dimensions ranging from 500 nm to 2 μm were prepared (Figures S1, S2). The grafting approach included three key stages (Figure 1b).

**Figure 1:**
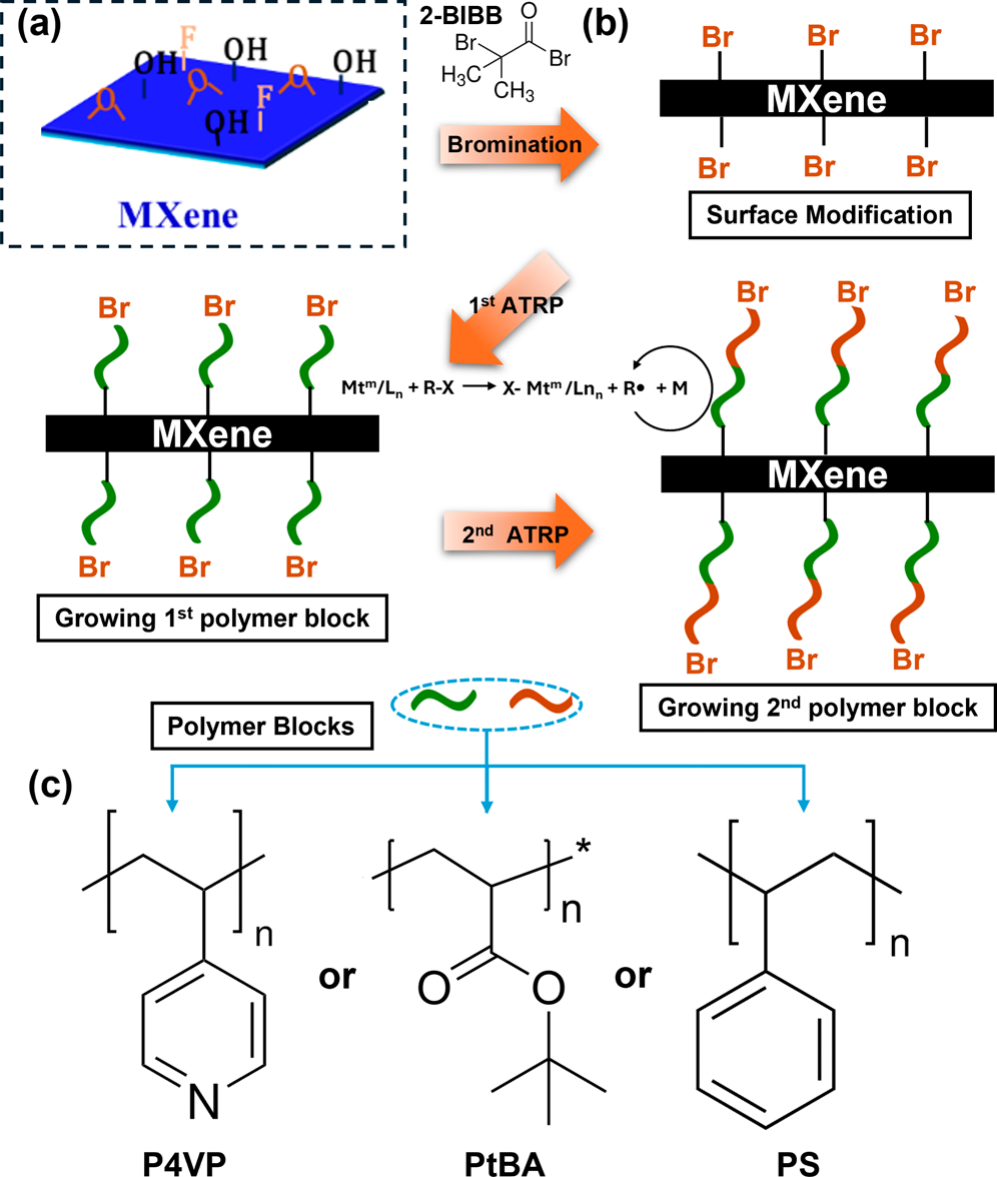
Synthesis of a MXene–polymer hybrids with an ATRP-based approach, starting from (a) pristine MXene through (b) three synthesis steps. The chemical equation presenting ATRP reaction is also displayed here, where Mt: transition metal, R: Polymer chain, X: Br or Cl, M: monomer. (c) Chemical structures of grafted polymers synthesized in this research.

First, the surface of the MXene was modified through a targeted functionalization reaction between surface hydroxyl groups and 2-bromoisobutyryl bromide (2-BIBB).^32^ This step converts the −OH terminations of the MXene to firmly anchored bromine-containing initiator sites, which are necessary for the subsequent ATRP process. This step occurs through a simple esterification reaction, establishing covalent linkages while maintaining the structural integrity of the MXene sheets without damaging their 2D morphology. Avoiding complex or custom-designed initiators, we employed a well-established and highly efficient ATRP initiator, 2-BIBB, which can directly react with the surface functional groups to form robust Ti–O–C bonds without complicated procedures.^33,34^ This enables precise and uniform polymer grafting without requiring additional synthetic steps to modify the initiator structure, distinguishing our approach from prior works.^17,18^

In the second stage, the growth of the first polymer block is initiated through the ATRP process via the reaction with a copper bromide (CuBr) catalyst (Figure 1b). The bromine-terminated surface sites serve as initiation points for controlled polymer chain growth, enabling the synthesis of well-defined polymer brushes with predetermined molecular weights. To fully utilize the capabilities of ATRP, we systematically varied key polymerization parameters, including reaction time, temperature, and monomer types, to understand the polymer growth kinetics and tailor molecular weight control as discussed below.

The final stage involves the growth of a second polymer block from the active chain ends of the first block, creating diblock copolymer architectures (Figure 1b). The living-polymerization nature of ATRP enables this sequential addition, maintaining control over both block lengths while preserving the original covalent attachment of the initiator to the MXene surface, with the freedom of choosing different polymer blocks regardless of sequence. Here, P4VP, PtBA, and PS were used to demonstrate the versatility of the synthetic approach (Figure 1c).

### Surface Functionalization of MXene Nanosheets

To achieve the proposed synthetic procedure, surface functionalization of the MXene sheets with ATRP initiator molecules is a crucial step that enables subsequent controlled polymer growth. The process involved a simple esterification reaction between surface – OH groups of MXene and typical ATRP initiator, 2-BIBB, establishing covalent ester linkages that serve as initiation sites (Figure 2a, eq 1):

1 $${\mathrm{Ti}}_{3}C_{2}T_{x}\text{−}\mathrm{OH}+\mathrm{Br}\text{−}C{\left({\mathrm{CH}}_{3}\right)}_{2}\text{−}C\left(\text{=}O\right)\text{−}\mathrm{Br}\to {\mathrm{Ti}}_{3}C_{2}T_{x}\text{−}O\text{−}C\left(\text{=}O\right)\text{−}C{\left({\mathrm{CH}}_{3}\right)}_{2}\text{−}\mathrm{Br}+\mathrm{HBr}$$

**Figure 2:**
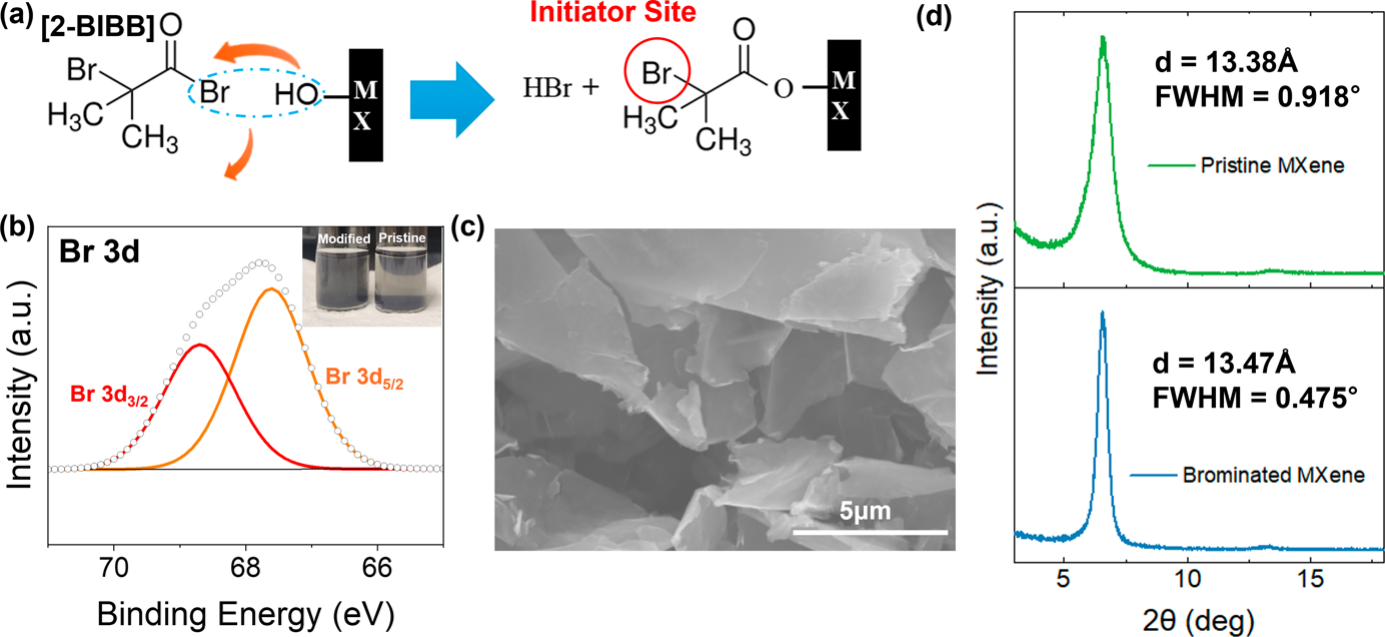
Surface modification of MXenes with ATRP initiator 2-BIBB. (a) Chemical structure of 2-BIBB and reaction schematics, (b) XPS Br 3d peak observed from the brominated MXene and solubility comparison with pristine MXene in acetone (inset), (c) SEM image of brominated MXene, and (d) WAXS spectra of pristine and brominated MXene.

In this reaction scheme, the acyl bromide group (C=O–Br) of 2-BIBB reacts with the hydroxyl groups on the MXene surface. During this process, the carbonyl carbon of 2-BIBB undergoes nucleophilic attack by the oxygen of the MXene’s hydroxyl group, forming a covalent ester linkage. Finally, the remaining Br on the attached 2-BIBB can function as an initiation site for various polymers. Here, a critical challenge in efficient functionalization or the surface stems from MXenes’ pronounced tendency to aggregate in nonaqueous solvents such as DMF, which is required for the bromination reaction. This aggregation occurs due to the loss of electrostatic repulsion and reduced surface charge when MXenes are transferred from their native aqueous environment to organic solvents. Such aggregation severely limits surface exposure, leading to heterogeneous initiator coverage and compromising the uniformity essential for controlled polymerization.

To address this fundamental obstacle, we developed a strategic sample preparation protocol that preserves maximum MXene surface area during the critical solvent exchange step. It includes first rapid cooling via liquid nitrogen prior to freeze-drying the MXene nanosheets. This freezing process creates a porous MXene structure by immobilizing water molecules between the sheets before aggregation can occur, essentially locking the sheets in their separated state. When the frozen water is subsequently removed through freeze-drying, the preserved spacing between sheets is maintained with a fluffy, sponge-like visual appearance that is extremely lightweight relative to its bulk volume, providing dramatically enhanced surface exposure during initial reaction compared to conventional processing methods, which could also be observed (Figure S3). This porous architecture enabled complete and uniform dispersion of MXene in DMF during the subsequent bromination step with 2-BIBB, as evidenced by significantly improved solubility in nonaqueous solvents and markedly enhanced Br signal intensity in XPS analysis. The effectiveness of this approach is further confirmed by the substantially higher initiator grafting efficiency achieved compared to common MXene processed without this aggregation prevention protocol, demonstrating that our method successfully overcomes the surface exposure limitations that have historically impeded controlled functionalization of MXenes in organic media, which is further described in the Experimental Section.

After the bromination, the attachment of 2-BIBB initiator molecules to the surface of MXene was verified through X-ray photoelectron spectroscopy (XPS) and energy-dispersive X-ray spectroscopy (EDX). High-resolution XPS spectra reveal the emergence of characteristic peaks corresponding to Br 3d_5/2_ and 3d_3/2_ doublet peaks at 67.6 and 68.7 eV, respectively, after initiator modification, confirming the presence of bromine-containing initiator molecules on the MXene surface (Figure 2b).^35,36^ Furthermore, in O 1s XPS spectra, the appearance of a distinct C=O peak at 533.8 eV originated from the ester bond of the 2-BIBB, an increment of the C–O peak at 531.7 eV, and a decrement of the −OH peak at 531.9 eV compared to pristine MXene due to replacement of the hydroxyl groups further supports successful attachment of the initiator (Figure S8).^37^

Additionally, a significant change in solvent compatibility was observed, as evidenced by the enhanced dispersibility of modified MXene sheets in acetone, which is known to be a relatively poor solvent for pristine MXenes due to low zeta potential.^38^ Indeed, while the pristine MXene exhibited rapid precipitation in acetone as expected, initiator-modified MXene maintained stable dispersion for several hours, indicating successful surface functionalization (Figure 2b inset). Meanwhile, the overall morphology of the MXene flakes was retained after the bromination process (Figure 2c, Figures S2–S5, S9). Finally, EDX analysis of the surface of the brominated flake confirmed the presence of bromine, with a characteristic peak at 1.48 keV (Figure S10).

Finally, wide-angle X-ray scattering (WAXS) analysis was performed before and after the bromination process to monitor any structural changes upon bromination (Figure 2d). The pristine MXene exhibited a characteristic (002) peak at 6.6° (corresponding to a *d*-spacing of 13.38 Å), while the brominated MXene showed a slightly shifted peak at 6.55° (13.47 Å), indicating a minor increase in *d*-spacing after surface modification.^39^ The absence of significant shifts or additional peaks confirmed that the structural integrity of the original MXene layers was preserved during a chemical reaction, with surface functionalization primarily occurring on the exposed outer surface. A substantial decrease in the full width at half-maximum (fwhm) from 0.92° to 0.475° can be attributed to prolonged heating (60 °C, 48 h at 60 °C, as described in Experimental Section) of MXene sheets during the bromination process, likely leading to improved stacking of the MXene sheets via an annealing effect.^40^ Additionally, the replacement of randomly arranged water molecules with grafted organic molecules is expected to result in more regular spacing. These findings collectively verify the successful surface modification while maintaining the original layered structure of MXene, ensuring its suitability for subsequent surface-initiated polymerization steps.

### Synthesis and Grafting of the First Polymer Block

Following the successful surface modification of MXene nanosheets with bromine-containing initiators, ATRP was employed to grow polymer chains from the surface (Figure 1). This approach enables controlled radical polymerization through a dynamic equilibrium between dormant and active species, where a copper-based catalyst system mediates the reversible transfer of halogen atoms. To demonstrate the versatility of this approach, three different monomers with distinct polarities were selected: 4-vinylpyridine (4VP), *tert*-butyl acrylate (tBA), and styrene (Figure 1c). Among these components, 4VP represents a highly polar monomer containing a pyridine ring, tBA offers moderate polarity through its ester group, while styrene provides nonpolar polymer chains with its aromatic ring structure.^41^ The obtained MXene–polymer hybrid materials with a single polymer block to the surface were named MXene-*g*-P4VP, MXene-*g*-PtBA, and MXene-*g*-PS, depending on the type of polymer grafted.

To confirm the controlled tunability of chain length achieved through the ATRP process, the synthesis was conducted at various time frames and temperatures, as reported in the existing literature, to observe an increase in molecular weight and length of each polymer chain with the varying variables.^31,42^ For example, MXene-*g*-P4VP hybrids with different lengths of surface-grown P4VP chains were synthesized using reaction times ranging from 5 min to 4 h and variable temperatures for fixed reaction times (for details see the Experimental Section), expecting an increase in polymer chain length at longer reaction times or higher reaction temperatures. After the first ATRP step was complete, the polymer growth on the surface of the MXene was analyzed, as discussed below (Figure 3).

**Figure 3:**
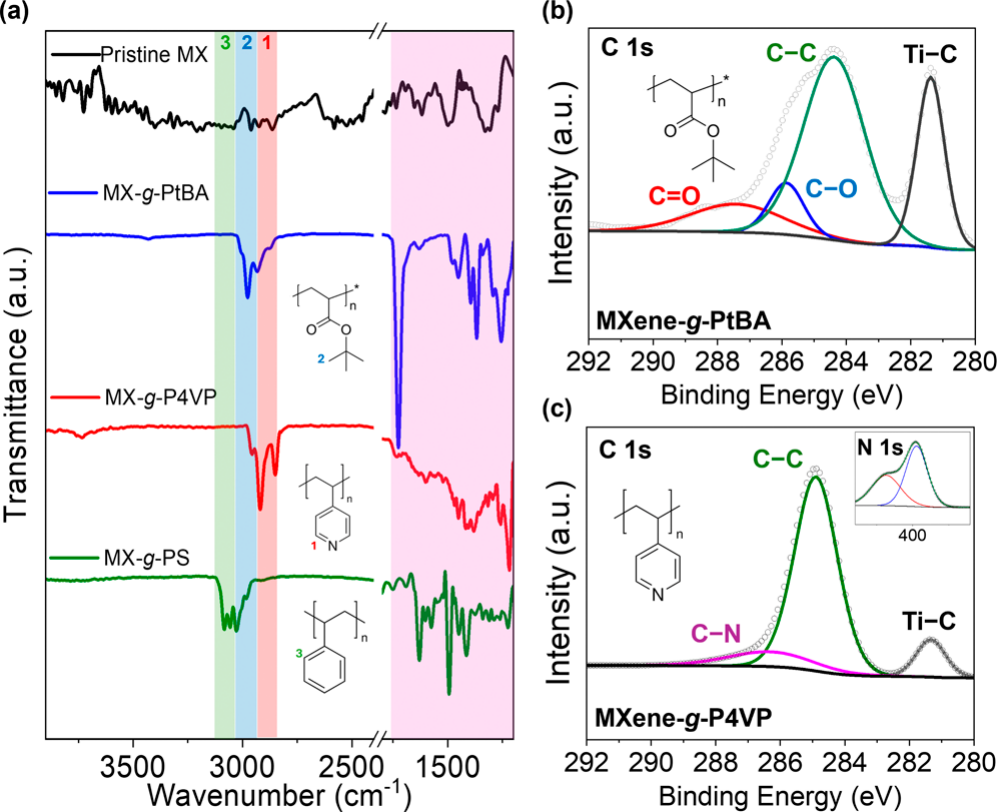
Chemical analysis of polymer grown on MXene surface using ATRP. (a) FT-IR plot of MXene–polymer hybrids after single block growth. Characteristic peaks of C–H stretching peaks at 2800 to 3200 cm^–1^ range are marked with color codes, XPS C 1s spectra of (b) MXene-*g*-PtBA and (c) MXene-*g*-P4VP samples with N 1s in the inset.

### Chemical Validation of the Polymer Block Grafting

First, it is important to note that the nature of MXenes typically inhibits traditional Fourier Transform Infrared (FTIR) spectroscopy due to their strong light absorption in the infrared region and complex surface chemistry (Figure S11).^43,44^ To resolve the issue, it was suggested that data correction methods and peak deconvolution approaches be applied to isolate and identify characteristic polymer signals.^41^ Although our samples with sufficiently thick polymer layers on the MXene surface allowed distinguishing FTIR peaks even before post-processing, well-acknowledged baseline deduction and flattening processes were used to obtain clearer characteristic signals confirming the presence and type of grafted polymer chains on the MXene surface. Indeed, the FTIR spectra revealed characteristic C–H stretching in the 2900–3100 cm^–1^ region, confirming the presence of grafted polymer chains with distinct chemical compositions (Figure 3a).^45^ For example, the MXene-*g*-PS sample exhibited peaks in the 3020–3100 cm^–1^ range, attributed to the aromatic C–H stretching vibrations of the benzene ring in polystyrene.^46^ The electron-withdrawing effect of the phenyl group increases bond stiffness, shifting the characteristic vibration to higher wavenumbers.

In contrast, the MXene-*g*-PtBA sample displayed FTIR peaks around 2950–2980 cm^–1^, corresponding to asymmetric stretching of aliphatic C–H bonds in the bulky *tert*-butyl group.^47^ The electronic effects of the branched *tert*-butyl substituent slightly elevate the vibration frequency compared to alkyl chains. Finally, the MXene-*g*-P4VP sample showed peaks in the 2900–2940 cm^–1^ range, originating from aliphatic C–H stretching in the polymer backbone and C–H stretching in the pyridine ring.^48^ Notably, the presence of nitrogen from the pyridine influences the C–H bonds, slightly lowering the stretching frequency. The samples also showed distinct characteristic peaks in the fingerprint region, including carbonyl (C=O) stretching vibration at 1730 cm^–1^ for PtBA and C–N stretching vibration at a lower wavenumber for P4VP.^49,50^ Here, the notably strong intensity of the C=O peak in MXene-*g*-PtBA, is expected to be resulting from the previously mentioned baseline correction required for FTIR analysis of MXene-based composites that involve concave baseline subtraction to isolate polymer signals from MXene’s strong background absorption. Importantly, complementary characterization, including XPS analysis, grafting density calculations, and morphological studies that will be discussed later, confirms consistent polymer coverage across all samples, indicating that the C=O peak intensity reflects analytical processing but not excessive surface coverage.

Next, XPS was conducted to confirm the successful grafting of PtBA and P4VP onto the MXene surface. The C 1s spectrum of MXene-*g*-PtBA exhibited distinct peaks corresponding to the chemical structure of PtBA, confirming the existence of the surface-grown polymers on MXene nanosheets (Figure 3b). While the prominent Ti–C peak at 281.3 eV was first observed, the dominant organic carbon peak at ∼ 284.8 eV was attributed to aliphatic C–C/C–H bonds from the *tert*-butyl group, followed by the ester C–O peak at ∼ 286.5 eV, and a weaker C=O peak observed at ∼ 288.5–289.0 eV.^35,51^ These characteristic spectral features align with the expected chemical composition of PtBA, confirming its successful grafting onto MXene.

For MXene-*g*-P4VP, the C 1s spectrum also displayed the characteristic aromatic C–C/C–H peak at ∼ 284.6 eV, corresponding to the pyridine ring backbone. A distinct C–N peak at ∼ 286.3 eV was detected, confirming the presence of nitrogen-bound carbon of the pyridine structure (Figure 3c).^52^ The electron-withdrawing effect of the pyridine nitrogen caused a shift in the C–N bonding energy, distinguishing it from typical aliphatic C–C bonds. Furthermore, the N 1s spectrum of MXene-*g*-P4VP exhibited distinct nitrogen peaks, which were absent for MXene-*g*-PtBA, where the peak positioned at approximately 399.0 eV can be assigned to the pyridine C–N distinguished from surface-adsorbed nitrogen from the atmosphere, further verifying the successful grafting of a nitrogen-containing polymer onto the MXene surface (Figure 3c inset).^53^ The clear presence of polymer-specific peaks in both C 1s and N 1s spectra provides further evidence of the precise and controlled nature of this surface-initiated polymerization strategy.

Meanwhile, distinct Br 3d peaks were observed after the polymerization steps, confirming the persistence of the ATRP initiator sites for the subsequent block growth, which will be utilized in the next step to grow the second polymer block (Figure S14).

Next, we moved on to investigate the bonding and surface stability of our MXene–polymer hybrids. First, the expected bonding stability of our grafted-from MXene–polymer hybrids, stemming from the robust covalent bonding established between polymer chains and MXene surfaces compared to conventional surface-adsorbed chains, was examined through a simple comparison experiment by exposing the composite to multiple solvent washing cycles (Figure 4a, b).

**Figure 4:**
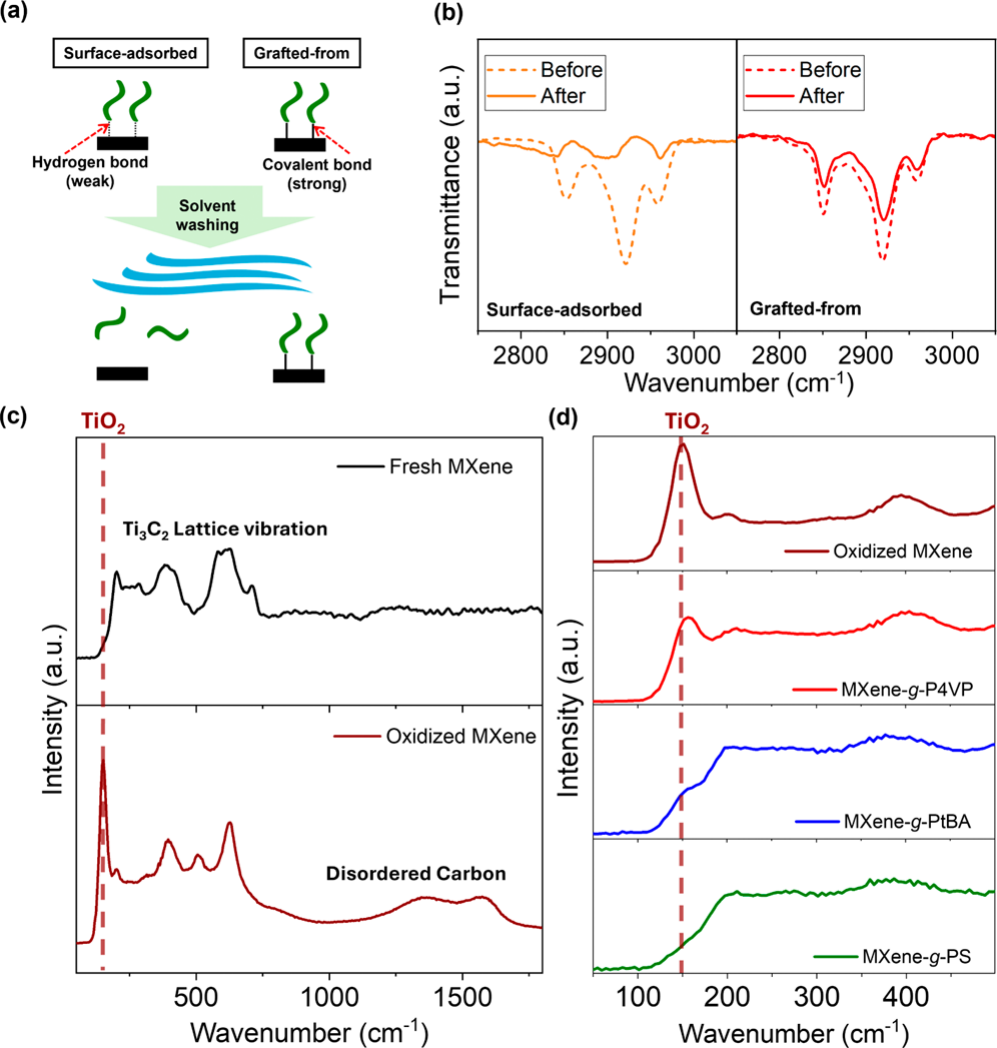
Bonding stability and surface oxidation protection property of our MXene–polymer hybrids. (a) Schematic describing the stability of strong covalent bonding via grafting-from approach and (b) comparison of characteristic FTIR peaks evolution of the MXene-*g*-P4VP samples produced by different fabrication methods before and after intense washing with good solvents. (c) Raman spectra comparison of pristine MXene before and after exposure to ambient air for 6 months and (d) Raman spectra comparison of pristine MXene, MXene-*g*-P4VP, MXene-*g*-PtBA, and MXene-*g*-PS samples after exposure to ambient air for 6 months. Raman peak position of TiO_2_ from oxidation at 155 cm^–1^ is marked with a brown dotted line.

Unlike conventional surface-adsorbed polymers that rely on weaker hydrogen bonding or van der Waals forces, the grafting-from approach explored in this study results in strong covalent bonds that maintain structural integrity even under harsh chemical conditions.^54,55^ This stability is demonstrated through the experiment described in Figure 4a, where MXene–polymer systems fabricated through different methods were subjected to multiple washing cycles with compatible solvents. For example, when the MXene-*g*-P4VP systems fabricated through our grafting-from approach and simple surface-adsorption process were exposed to multiple isopropyl alcohol (IPA) washing cycles, FTIR analysis revealed that while the characteristic C–H signals from P4VP chains were well observed for both grafted MXene-*g*-P4VP and surface-adsorbed MXene-P4VP samples, the signal was drastically reduced after solvent washing for the sample prepared via simple physical adsorption, indicating significant removal of P4VP chains from the surface (Figure 4b). In contrast, MXene-*g*-P4VP with covalently bonded grafted-from polymer maintained its characteristic peaks with minimal intensity loss, confirming the exceptional durability of the chemical interface under solvent treatment. This enhanced stability ensures consistent performance across varied solvent conditions and extends the applicability of the composite matrices.

Furthermore, we tested the MXene oxidation prevention functionality of the grafted polymer layer, which is one of the most critical challenges in MXene applications in composite materials. Raman spectroscopy was employed to monitor the oxidation state of MXene exposed to ambient air by detecting the formation of TiO_2_ characteristic peaks that accompany MXene oxidation.^56^ As expected, for Raman spectra of our pristine MXene before and after 6 months of ambient air exposure, the oxidized MXene sample exhibited a distinct TiO_2_ peak at 155 cm^–1^ with increased disordered carbon region peaks from 1200 cm^–1^ to 1700 cm^–1^, confirming significant oxidation of unprotected MXene under ambient conditions (Figure 4c).

Since the grafted polymer chains in our MXene–polymer hybrids also contain obvious carbon-related Raman peaks due to the inherent carbon chain, which overlap with disordered carbons, we focused specifically on monitoring the TiO_2_ peak at 155 cm^–1^ as the primary indicator of MXene transformations. Specifically, pristine MXene and our polymer-grafted samples (MXene-*g*-P4VP, MXene-*g*-PtBA, and MXene-*g*-PS) were exposed to ambient air for 6 months, and the TiO_2_ presence was evaluated (Figure 4d).

As we observed, all polymer-grafted MXenes showed significant suppression of the TiO_2_ peak formation compared to pristine MXene, demonstrating that the densely grafted polymer brushes effectively protect the MXene surface from oxidation. Interestingly, the degree of protection also varied with the types of grafted polymer chains based on their hydrophobicity. For example, MXene-*g*-P4VP, in which the MXene is covered by relatively hydrophilic polymer, P4VP, still exhibited some extent of MXene oxidation. In contrast, MXene-*g*-PtBA showed more suppressed TiO_2_ formation, while MXene-*g*-PS demonstrated almost complete prevention of TiO_2_ formation. The result can be explained by the hydrophobic property of PtBA and PS to repel moisture and oxygen from the air from reaching the MXene surface.^57^ This tunable oxidation resistance implies another functional advantage of our surface-grafted MXene–polymer hybrids via SI-ATRP, which enables facile incorporation of various polymer blocks with different characteristics, demonstrating selective modification of MXene’s surface property with practical benefits for long-term stability under ambient conditions.

### Molecular Weight Control and Characterization of Polymer Brushes

Next, we explored the ATRP grafting-from approach’s capability to precisely control the length of the polymer chains with high uniformity and narrow molecular weight distribution, which are directly inherited from the ATRP process and facilitated tailored molecular weight, consistent surface coverage, polymer layer thickness, and brush density.^58^

Considering the nature of the MXene–polymer system, which consists of micrometer-scale 2D nanosheets with a covalently bound polymer layer, which inhibits the direct molecular weight measurement of the polymer brushes through conventional gel permeation chromatography (GPC). Therefore, we adopted an ester cleavage process using hydrolysis, which selectively breaks the Ti–O–C bond between MXene nanosheets and initiator 2-BIBB (Figure 5a).^32^ After the cleavage, we were able to measure free, isolated polymer chains detached from the MXene surface using GPC analysis. For example, when P4VP chains grown for 30 and 240 min with the fixed reaction temperature of 40 °C were cleaved from MXene and characterized, peaks shifted to shorter permeation times corresponding to molecular weight increase from 45 600 g/mol to 137 000 g/mol, which corresponds to approximate degree of polymerization (DP) value of 434 to 1304, respectively (Figure 5b).

**Figure 5:**
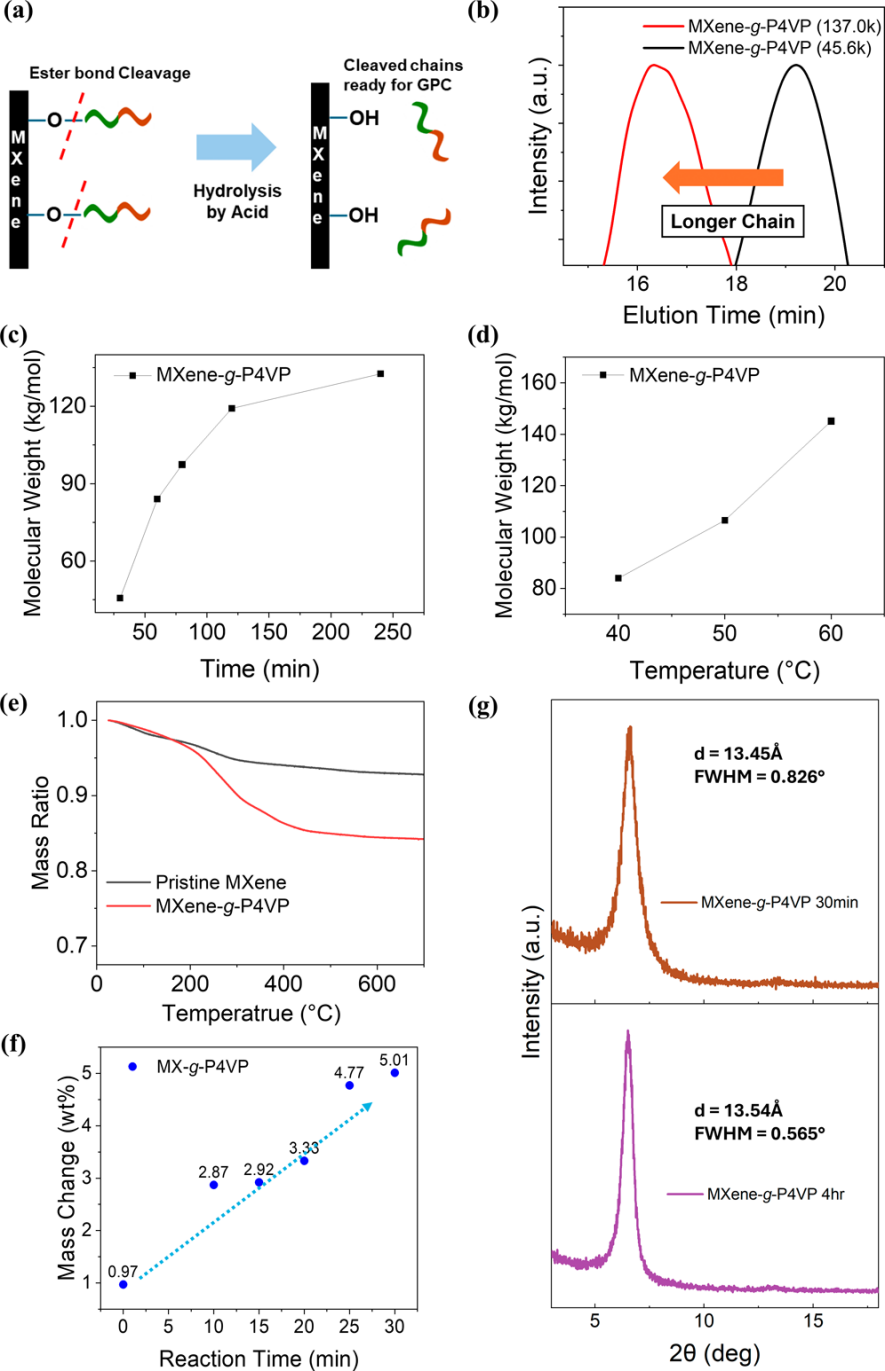
Molecular weight characterization of cleaved ATRP-grown polymers on the MXene surface. (a) Cleavage process for characterization of polymer chains, (b) GPC peak shift of P4VP chains grown for 30 min to 4 h, (c) GPC-measured molecular weight of P4VP chains over different reaction times, and (d) reaction temperatures. TGA plot of Pristine MXene and MXene-*g*-P4VP systems is shown on (e), followed by plots of (f) decreased weight ratio by TGA analysis for MXene-*g*-P4VP with different synthesis durations and (g) WAXS data comparison of MXene-*g*-P4VP samples reacted for 30 min and 4 h.

The polydispersity index remained below 1.2 throughout the samples, indicating expected uniformity of grown polymer chains via ATRP in contrast to traditional polymerizations. Through this method, we obtained the time-differentiated molecular weight of the cleaved P4VP and PtBA chains, confirming the consistency of the chain growth with increased reaction time (Figure 5c, S15a). Furthermore, the molecular weight graph indicates that by varying the reaction temperature from 40 to 60 °C, the molecular weight of the P4VP chain rises from 84 000 g/mol to 145 100 g/mol, corresponding to DP of 800 to 1382, due to the acceleration of polymer growth (Figure 5d).

In addition, thermogravimetric analysis (TGA) was performed, with a particular focus on shorter polymer chains that are less detectable by GPC. As expected, the TGA showed distinct weight loss regions corresponding to the degradation of grafted polymer chains while the MXene phase was preserved (Figure 5e). For the MXene-*g*-P4VP sample with 30 min of reaction time, the initial weight loss below 180 °C can be attributed to the removal of surface-adsorbed water and residual solvent, while the significant weight loss of approximately 5.0 wt % between 300 and 450 °C corresponds to the thermal decomposition of grafted P4VP polymer.^47^ The polymer content derived from TGA data exhibits a consistent increase over polymerization time. For instance, the polymer weight fraction increased from 2.87 wt % to 5.0 wt % with extended polymerization time from 10 to 30 min for P4VP, further correlating with the GPC data (Figure 5f). The same trend was also observed for the MXene-*g*-PtBA samples (Figure S15b).

Finally, WAXS analysis of MXene-*g*-P4VP samples with varying reaction times was conducted to examine the effect of polymer growth on the interlayer spacing of the MXene phase (Figure 5g). The diffraction patterns for MXene-*g*-P4VP samples, produced at reaction durations of 30 min and 4 h, consistently exhibited the characteristic (002) peak at 6.57° (*d*-spacing ∼ 13.45 Å) and 6.52° (*d*-spacing ∼ 13.54 Å), respectively. These *d*-spacings are close to those of the initial brominated MXene nanosheets, indicating minimal expansion during surface polymerization. Interestingly, we also observed a noticeable decrease in the full width at half-maximum of the (002) peak between the 30 min and 4-h samples, likely due to a thermal annealing effect that promotes uniformity of modified nanosheets, as observed during the bromination process. Again, we assume the prolonged heating during the polymerization step led to partial alignment of the MXene sheets, resulting in a more ordered layered structure. Overall, this observation confirms that polymer growth is mainly limited to the surface of MXene nanosheets.

### Synthesis of Second Polymer Block for Diblock Shells

Upon successful growth of single polymer blocks on MXene surfaces, we conducted a second ATRP reaction on the functionalizing nanosheets to create diblock copolymer chains as previously described (Figure 1b). Rigorous purification of the MXene–polymer systems was performed before and after each synthesis step to eliminate any residual monomers from the synthesis of the first block to avoid additional polymerization during the process.

To demonstrate the versatility of this ATRP-based approach, we successfully synthesized various diblock combinations with different block sequences and components. Specifically, successful synthesis was achieved for MXene-*g*-P4VP-*b*-PtBA, MXene-*g*-P4VP-*b*-PS, and MXene-*g*-PtBA-*b*-PS systems, as illustrated in Figure 6a, highlighting the method’s flexibility in controlling block composition and sequence.

**Figure 6:**
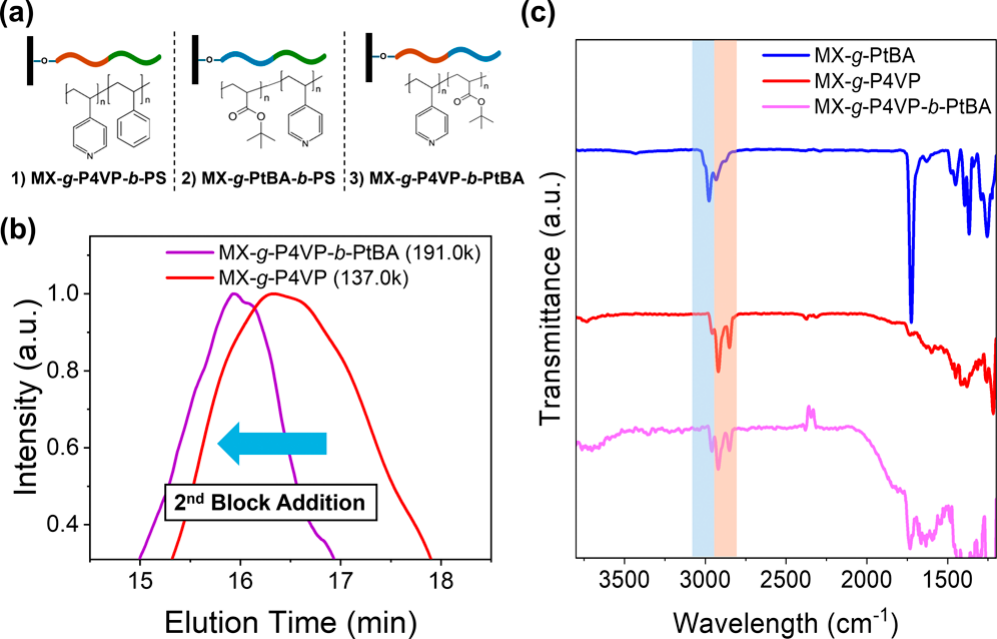
Diblock copolymer synthesis on the surface of the MXenes. (a) Schematic and chemical structure of synthesized MXene-Diblock copolymer systems, (b) GPC peak shift after growing an additional PtBA block on MXene-*g*-P4VP to form MXene-*g*-P4VP-*b*-PtBA, and (c) FT-IR spectra comparison of samples after diblock synthesis. Tinted bars indicate inheritance of specific polymer peaks to the diblock copolymer.

For verification of the growth of the second polymer block, we first cleaved polymer chains from the partial amount of MXene sheets grafted with the first polymer block. Then we analyzed the cleaved brushes with GPC to determine the initial molecular weight before growing the second block. The remaining MXene–polymer hybrid was then subjected to a second stage of the ATRP reaction for the growth of the second polymer block. Following this step, another cleavage and GPC analysis was performed to study the differences in molecular weights of the polymer brushes, providing direct evidence of successful second block growth.

As shown in Figure 6b, the GPC-measured molecular weight of polymer chains for the MXene-*g*-P4VP and MXene-*g*-P4VP-*b*-PtBA diblock copolymer system shifted to 191 000 g/mol from 137 000 g/mol after the growth of the PtBA second block. Thus, it can be confirmed that the second block was grown from the end of the initial P4VP block, forming the desired MXene-*g*-P4VP-*b*-PtBA heterostructure with diblock copolymer brushes.

FTIR spectroscopy also confirmed diblock copolymer formation with distinct spectral signatures for each block (Figure 6c). The FTIR spectra of the MXene-*g*-P4VP-*b*-PtBA system showed the characteristic C–H stretching vibrations from both polymer blocks in the 2900–3100 cm^–1^ region. Specifically, the spectra displayed aromatic C–H stretching peaks at 3020–3100 cm^–1^ from the PS block and aliphatic C–H stretching at 2900–2940 cm^–1^ from the P4VP block. Similarly, for MXene-*g*-PtBA-*b*-PS, the FTIR spectra exhibited the characteristic C–H stretching at 2950–2980 cm^–1^ from the *tert*-butyl groups of PtBA alongside the aromatic C–H stretching of PS at higher wavenumbers (Figure S16) with the same trend followed by MXene-*g*-P4VP-*b*-PS samples (Figure S17). In all cases, the FTIR spectra of the diblock copolymers showed a superposition of the characteristic peaks from the individual blocks, further validating the sequential growth of the second polymer chains while maintaining the first block. These spectroscopic observations and GPC results validate the synthesized diblock copolymer architectures on MXene surfaces, allowing for precise control over composition and structure.

The XPS analysis further corroborated these findings. As previously mentioned, when examining the Br 3d spectra of MXene-*g*-P4VP-*b*-PtBA synthesized using CuBr as a catalyst, we observed that bromide signals were still detectable, albeit at reduced intensity compared to the MXene-*g*-P4VP initiator (Figure S14). The preservation of these terminal bromide groups after the second polymerization step indicates the living nature of the polymerization process and suggests the possibility of further chain extension to create triblock copolymers in future studies. This observation confirms the preferable location of terminal groups at the topmost surface, thus confirming the ‘brush regime’ as will be further discussed in the next section.

### Surface Morphology of MXene–Polymer Hybrid Nanosheets

It is worth noting that due to the low contrast of polymer shells compared to the metallic element-based MXene, transmission electron microscopy (TEM) analysis of modified MXene nanosheets is challenging (Figure S18). In contrast, atomic force microscopy (AFM) offers 3D topographical features and enables quantitative relationships between polymer chain length and layer thickness, providing critical insights into the polymer regime on MXene surfaces.^59,60^

AFM topography of pristine and brominated MXene flakes revealed that both maintained intrinsic 2D morphology with smooth surfaces and thicknesses of approximately 2 to 3 nm, indicating that the bromination process preserved the structural integrity of the nanosheets (Figures S2, S4). After the polymerization, a significant change in morphology was observed as polymer shells grew on the MXene surfaces (Figure 7). As mentioned earlier, the AFM images showed clear morphological characteristics of the polymer-functionalized MXene flakes. These flakes retained their structure with lateral dimensions between 500 nm and 2 μm, common sizes of the original MXene flakes utilized as starting materials (Figures 7, S2, S18–S20).

**Figure 7:**
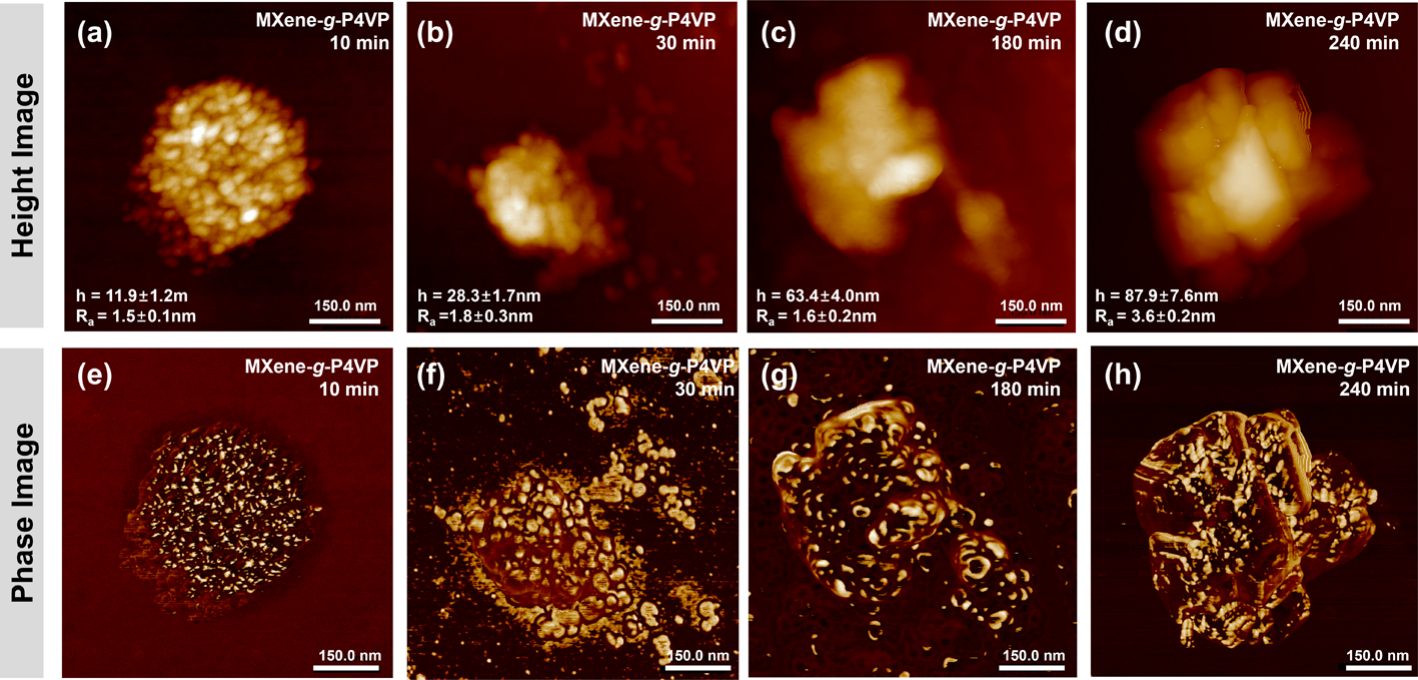
Surface morphology of MXene-*g*-P4VP nanosheets characterized by AFM. Evolution of topographical images MXene-*g*-P4VP nanosheets after polymerization for (a) 10, (b) 30, (c) 180, and (d) 240 min are shown, and corresponding phase images ((e), (f), (g), and (h), respectively). Mean height and R_a_ values are provided on the height images.

The AFM topography of both MXene-*g*-P4VP and MXene-*g*-PtBA samples acquired over larger scanning areas demonstrated that multiple flakes exhibit consistent morphology and thickness, validating the uniformity and reproducibility of the ATRP polymerization approach across diverse MXene sheets (Figure S19). Moving on to a detailed analysis of the obtained AFM images, MXene-*b*-P4VP with a 10 min reaction time showed a total thickness of 11.9 ± 1.2 nm (Figure 7a). Considering that in mixed suspension, the polymer layer grows on both sides of MXene flakes and accounting for the thickness of the original MXene flake (2 to 3 nm), the single-side polymer shell thickness is calculated to be 4.5 ± 0.6 nm. Using the same approach, MXene-*b*-PtBA synthesized with a 1-h reaction time showing a single side thickness of 14.3 ± 1.6 nm (calculated from a total double thickness of 31.6 ± 3.1 nm) (Figure S20). The height profiles extracted from multiple line scans across individual flakes confirm the uniform thickness distribution, along with a further smoothing effect as the chain length increases (Figure S21).

Next, we considered an expected systematic increase in polymer layer thickness that correlated directly with polymerization time. For instance, for the P4VP samples, the calculated single-side thickness increased from 4.5 ± 0.6 nm at 10 min to 42.5 ± 3.8 nm at 4 h, showing a linear relationship between polymerization time and brush thickness (Figure 7, Table S1). Importantly, the topography images reveal that polymer growth fully covers the surface of the MXene flakes, as confirmed by low phase contrast, while adding to their overall thickness. Simultaneously, despite the significant change in polymer thickness to the corresponding synthesis conditions, the surface roughness value (R_a_) consistently remained below 10% of the total thickness for all samples, with representative R_a_ values ranging from 1.5 ± 0.1 nm for the 10 min P4VP sample to 3.6 ± 0.2 nm for the 4-h sample (Figure S22). This consistently low roughness-to-thickness ratio indicates uniform polymer layer growth regardless of chain length.

Furthermore, by combining the polymer layer thickness data from AFM and molecular weight obtained from GPC, we can estimate grafting density to evaluate the internal morphology of growing polymer chains. As is known, the grafting density of the polymer brushes on the MXene surface can be calculated using the following equation:

2 $$GraftingDensity\left(chains/nm^{2}\right)=\left(h\times N_{A}\times \rho \right)/MW$$

where *h* represents the layer thickness, *N*_*A*_ is Avogadro’s number, ρ is the polymer density (0.95–1.15 g/cm^3^ for the polymers used), and *MW* is the molecular weight, as measured by GPC.^61^

The calculated grafting densities across multiple samples were consistently in the 0.18–0.25 chains/nm^2^ range, comparable to values reported for grafted-from polymer layers on uniform surfaces (Table S1). Combined with a radius of gyration of the polymer brushes for a given molecular weight, the value indicates that our MXene–polymer system is in a ‘brush regime’. The brush regime refers to a high grafting density state where polymer chains are sufficiently close to induce strong steric repulsions, forcing them into stretched conformations perpendicular to the substrate surface. This contrasts with the ‘mushroom regime’ (low grafting density), where chains adopt random coil configurations with minimal interchain interactions.^62,63^

The brush growth regime is characterized by a linear relationship between layer thickness and molecular weight (*h* ∝ MW), as opposed to the mushroom regime, where sparsely grafted chains adopt a random coil configuration with thickness scaling as *h* ∝ MW^(v)^ where *v* < 3/5 is a theoretical expectation for random coils and mushroom regimes.^64^ A linear relationship was observed across the entire molecular weight range studied by plotting thickness against molecular weight for all measured samples from AFM and GPC data (Figure 8a).

**Figure 8:**
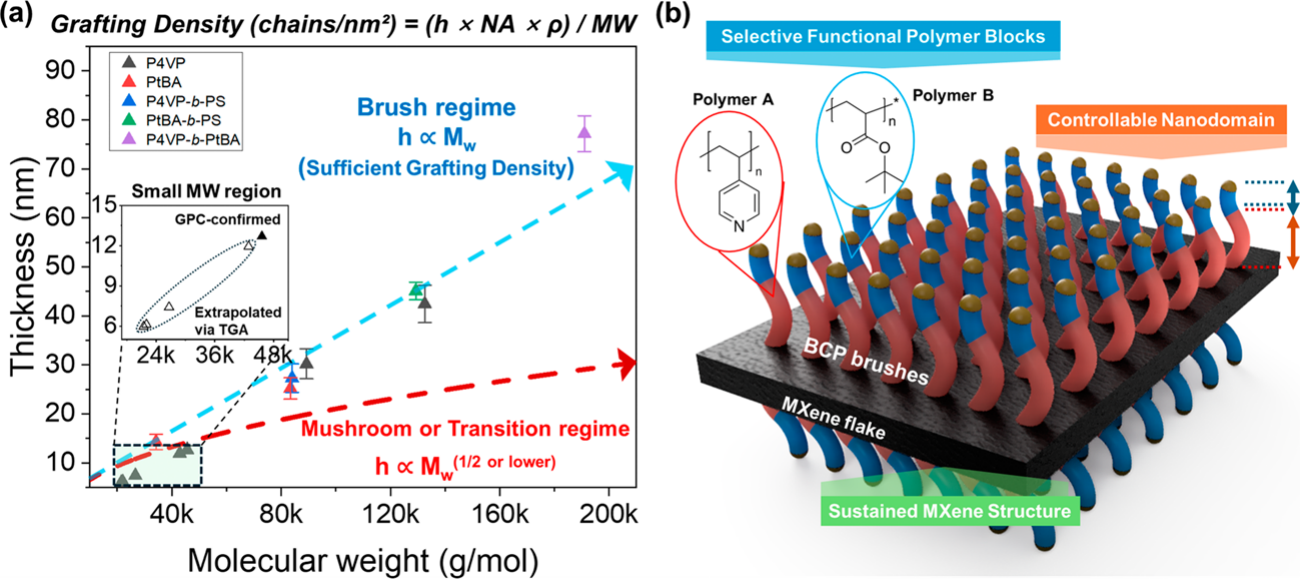
(a) Thickness of polymer layer vs molecular weight plot of MXene–polymer samples from GPC and AFM data. The blue dotted line describes the expected trend for the brush regime, and the red dotted line is for the mushroom or transition regime. The inset shows a small molecular weight region where the molecular weight and thickness of given polymer chains were extrapolated from the trend using TGA data. The equation driving grafting density from the given plot is also depicted above. (b) Schematic drawing showing the structure and key advantages of the final product.

The brush regime directly confirmed the linear dependency between thickness and molecular weight. This regime is the defining characteristic of polymer chains that are densely grafted, causing steric repulsion forces to extend them mostly perpendicular to the surface. This contrasts with the mushroom or transition regimes, where thickness would scale with molecular weight according to a different power law (for example, here, *h* ∝ MW^(1/2)^). Furthermore, using the established linear relationship between molecular weight and AFM-measured height, we could extrapolate molecular weights for samples with much shorter chains, for which only TGA data were available. This extrapolation further validates the brush regime for ATRP grafting-from on MXene surfaces and provides a predictive trend for estimating molecular weights of ultrathin polymer layers that fall below the detection limits of conventional GPC (inset of Figure 8a).

The AFM images also revealed a clear morphological evolution, where the polymer layer’s appearance transitioned from a cloud-like, grainy texture to a more uniform surface as chain length and thickness increased, which can also be observed in the height images. This observation supports the development of more densely packed and extended polymer brush configurations at higher molecular weights with uniform surface topography. The images also revealed a clear morphological evolution, where the polymer layer’s appearance transitioned from a cloud-like, grainy texture to a more uniform surface as chain length and thickness increased (Figure S21). This further supports densely packed and extended polymer brushes at higher molecular weights with uniform surface topography.

The observed brush regime structure of our MXene–polymer hybrids implies significant features closely related to functional characteristics of the system. In this state, where polymer chains are in the form of dense, stretched brushes perpendicularly rising from the surface, uniform surface coverage and maximized accessibility of the existing functional groups on the polymer are granted. This well-defined feature is critical for precisely controlling the morphology interaction of the polymer matrices with external systems, which is a distinct advantage compared to traditional physically absorbed layers with hard-to-configure random chain orientation.^53,59^

In addition, beyond the controlled synthesis of well-defined polymer architectures, we also found promising signs of self-assembly behavior of diblock copolymer in the obtained MXene–polymer hybrid systems. When a MXene-*g*-P4VP-*b*-PS sample with a total thickness of approximately 40 nm was exposed to toluene vapor, which is a well-known solvent for preferential swelling of PS blocks, the emergence of dendrite-like morphological features along with a thickness increase to 135 nm was observed (Figure S23).^65^ This observation suggests that covalently attached diblock copolymers retain their assembly capabilities even when tethered to the MXene surface. This spontaneous organization could be utilized to generate hierarchical surface patterns with customized domain sizes due to confined microphase separation.

Overall, the significant advantages of our MXene–polymer hybrid system can be summarized into three distinct features (Figure 8b). First, the freedom to grow selective functional polymer blocks provides highly customizable surface chemistry for targeted interactions with specific environments. Second, the controllable nanodomains enable the precise spatial arrangement of polymer brushes with specific functional groups at the nanoscale level. Lastly, the sustained MXene’s 2D structure maintains the inherent electronic and mechanical properties of the 2D nanosheets, even after extensive surface modification through this process. This demonstrates that our grafting-from approach offers the benefits of both the stability of covalent attachment and the preserved ability of block copolymers to self-organize into complex nanostructures.

## Conclusions

This study demonstrates ATRP-based covalent surface modification of Ti_3_C_2_T_*x*_ MXene, enabling precise control over the polymer architecture and composition with comprehensive fundamental analysis, filling the intellectual gap in the surprisingly underexplored combination of the two components. Based on the grafting-from methodology, including specialized procedures to address inherent challenges in adopting SI-ATRP for MXene, we synthesized a series of well-defined MXene–polymer hybrids, including MXene-*g*-P4VP, MXene-*g*-PtBA, and MXene-*g*-PS homopolymer systems with varying chain lengths via synthesis time and temperature control. We synthesized diblock copolymer architectures on MXene–polymer surfaces, yielding MXene-*g*-P4VP-*b*-PtBA, MXene-*g*-P4VP-*b*-PS, and MXene-*g*-PtBA-*b*-PS hybrids with accurate and predictable block sequences and compositions, the detailed study of which in the integration of SI-ATRP and MXenes has not been demonstrated until now.

The key advantage of the developed ATRP-based grafting-from approach over physical mixing or adsorption methods is the covalent bonding between polymer chains and MXene. While conventional approaches rely on relatively weak intermolecular forces, our method yields covalent bonds that ensure structural integrity even under harsh environmental conditions. The strong bond of the polymer brushes to the MXenes’ surface was validated through solvent resistance tests, with preserved FTIR signals from polymer chains even after several solvent washes, compared to physically adsorbed polymer chains, which were easily detached from MXene surfaces in control samples.

Additionally, the covalently grafted polymer layers provided tunable functional benefits for adjusting interfaces with surrounding polymer matrices, enhancing the oxidation resistance of MXene, with the degree of protection depending on the selection of grafted polymers. Such uniform and dense polymer coverage on MXene surfaces enables reliable interfacial robustness and offers versatile avenues for tailoring surface chemistry and properties. Furthermore, the brush architecture was confirmed by morphological characterization, which exhibits specific characteristics matching those of previously studied polymer brushes. It ensures consistent polymer grafting across individual MXene surfaces, allowing for robust surface functionality and diverse surface chemistry and composition.

Moreover, the diblock copolymer coating offers several distinct advantages, including the ability to create multifunctional interfaces where each polymer block may contribute different properties, the incorporation of stimuli-responsive elements through appropriate block selection, and the facilitation of tailored interactions with surrounding media through the selective exposure of grafted blocks that may further enhance discovered functionalities. The versatility demonstrated by the synthesis of multiple diblock combinations (P4VP-*b*-PtBA, P4VP-*b*-PS, and PtBA-*b*-PS) highlights the robustness of this approach and its potential for creating more complex tunable MXene–polymer hybrids. Tailoring the surface initiators accordingly in the first surface functionalization step is a surface modification platform for SI-ATRP of other living or controlled polymerization techniques, such as reversible addition–fragmentation chain transfer (RAFT) polymerizations, opening even further opportunities for broader polymer architectures and functionalities of 2D nanosheets.

## Experimental Section

### Materials

Anhydrous Dimethylformamide (DMF, 99.8%), *N*-Methyl-2-pyrrolidone (NMP, 99.5%), 2-Bromoisobutyrl Bromide­(2-BIBB, 998%), Ethyl a-bromoisobutyrate (EBIB, 98%), Copper Bromide (CuBr, 98%), Copper Chloride (CuCl, 97%), N,N,N,N,N-Pentamethyldiethylenetriamine (PMDETA, 99%), Tris­[2-(dimethylamino)­ethyl]­amine (Me6TREN, 97%), 4-vinylpyridine (4VP, 95%, contains 100 ppm hydroquinone as inhibitor), *tert*-butyl acrylate (tBA, 98%, contains 10–20 ppm monomethyl ether hydroquinone as inhibitor), styrene (≥99%), isopropyl alcohol (IPA, anhydrous, 99.5%), 2-butanone (≥99.0%), and anisole (anhydrous, 99.7%) were purchased from Sigma-Aldrich and used as received unless otherwise specified. Ti_3_AlC_2_ MAX phase for MXene synthesis was purchased from Carbon Ukraine.

### Synthesis of Ti_3_C_2_T_*x*_ MXene

In this study, we used delaminated Ti_3_C_2_T*_*x*_* MXene nanosheets as synthesized through a selective wet-chemical etching process that removed aluminum from the Ti_3_AlC_2_ MAX phase from Carbon Ukraine, Ltd.^1^ MXene flakes were extracted from a concentrated sediment containing delaminated MXene and redispersed in ultrapure deionized water, hand-shaken, and centrifuged for 1 h at 3500 rpm. The initial colloidal solution contained 0.0042 wt % MXene after preparation. Two separate MXene solutions were prepared by centrifuging 5 μL of 0.0042 wt % MXene solution at 3500 rpm and extracting the supernatant until a 0.5 wt % concentration of MXene in water was reached. All specimens were stored at −80 °C before use to maintain stability and prevent oxidation.

### Preparation of Ti_3_C_2_T_*x*_ MXene for Surface Functionalization

Before applying freeze-drying to remove water for surface functionalization of MXene, rapid cooling via liquid nitrogen was adopted to ensure MXene flakes maintain high surface exposure for efficient surface modification through inhibiting the sheets from aggregating during the required solvent exchange process. Briefly, in a falcon tube, the concentrated MXene solution was dispersed in DI water with a concentration of 3 mg/mL. The sample was then rapidly frozen by placing the tube into a vessel filled with liquid nitrogen to let water freeze without letting the flakes to stagger, followed by freeze-drying to remove the frozen water while maintaining space between each flake.

### Surface Bromination of Ti_3_C_2_T_*x*_ MXene

The modification was achieved through simple mixing of DMF-dispersed MXene with 2-BIBB in the presence of NMP to neutralize the resultant byproduct HBr, without the need for additional catalysts or complex reaction conditions. Briefly, in a round-bottom flask, 100 mg of freeze-dried MXene flakes were dissolved in 30 mL DMF, followed by adding 30 mL of NMP while stirring. The flask was sealed and purged with Ar for at least 1 h with continuous stirring. After the purging is complete, 25 mL of 2-BIBB was slowly added to the solution, followed by heating to 60 °C. The reaction was conducted for 48 h, and the resulting solution was vacuum-filtered and washed with acetone at least 3 times to remove solvents and any unreacted 2-BIBB initiators to achieve bromine-functionalized MXene sheets (named as MXene-Br).

### Synthesis of MXene-*g*-P4VP, MXene-*g*-PtBA, and MXene-*g*-PS Single Blocks

The MXene-Br was used as a macroinitiator for surface-initiated ATRP. In a typical procedure, MXene-Br was dispersed in the corresponding solvent and degassed by inert gas exchange for 1 h. CuBr catalyst and monomer (with specified amounts shown below) for each sample were added under an inert atmosphere. The reaction mixture was then heated to 60 °C and stirred for predetermined times to achieve controlled polymer growth.

For MXene-*g*-P4VP, in a 100 mL pressure vessel, 25 mg of MXene-Br was dispersed in 10 mL isopropanol (IPA) with a short sonication time of 3 min to enhance dispersion. After the flakes were well-dispersed, 0.05 mmol of CuBr and an equal molar amount of PMDETA were added, followed by 25 mmol of 4-vinylpyridine (4VP) monomer. The vessel was then sealed tightly and purged with Ar for at least an hour while stirring to remove oxygen. The polymerization was conducted at desired temperatures of 40 °C, 50 °C, and 60 °C under Ar gas. The reaction was stopped at predetermined times (30–240 min) with continuous stirring to achieve different polymer chain lengths. After the desired time was reached, the reaction was quenched by placing the vessel in ice, followed by the purification steps stated below. For the MXene-*g*-PtBA and MXene-*g*-PS, the overall procedure was the same as MXene-*g*-P4VP synthesis, except that 2-butanone and *tert*-butyl acrylate (tBA) were used for solvent and monomer for PtBA, and anisole and styrene were used for PS.

### Synthesis of MXene-*g*-P4VP-*b*-PS, MXene-*g*-PtBA-*b*-PS, and MXene-*g*-P4VP-*b*-PtBA Diblocks

#### Synthesis of MXene-*g*-P4VP-*b*-PtBA Diblock Copolymer

In a typical procedure, 20 mg of purified MXene-*g*-P4VP composite (with predetermined P4VP chain length) was dispersed in 8 mL of 2-butanone in a 50 mL pressure vessel using brief sonication to ensure uniform dispersion. After achieving homogeneous dispersion, the vessel was sealed and purged with argon for 60 min with continuous stirring to remove oxygen. Subsequently, 0.04 mmol of CuBr catalyst and an equimolar amount of PMDETA ligand were added to the reaction vessel under an inert atmosphere. Following catalyst addition, 20 mmol of *tert*-butyl acrylate (tBA) monomer was introduced to the reaction mixture. The vessel was resealed, and the polymerization was conducted at 60 °C for 2 h under continuous stirring. After polymerization, the reaction was quenched in an ice bath to stop further growth. The resulting MXene-*g*-P4VP-*b*-PtBA diblock copolymer was purified following the procedure described in the purification section.

#### Synthesis of MXene-*g*-P4VP-*b*-PS Diblock Copolymer

To synthesize MXene-*g*-P4VP-*b*-PS diblock copolymer, 20 mg of purified MXene-*g*-P4VP composite was dispersed in 8 mL of anisole by sonication. After achieving uniform dispersion, the vessel was sealed and degassed with Ar for 60 min. Under an inert atmosphere, 0.04 mmol of CuBr and an equimolar amount of PMDETA were added, followed by 20 mmol of styrene monomer. The polymerization was conducted at 90 °C for 4 h. After polymerization, the reaction was quenched in an ice bath to stop further growth, followed by standard purification procedures.

#### Synthesis of MXene-*g*-PtBA-*b*-PS Diblock Copolymer

To synthesize the MXene-*g*-PtBA-*b*-PS diblock copolymer composite, 20 mg of purified MXene-*g*-PtBA composite was dispersed in 8 mL of anisole. After degassing the mixture with Ar for 60 min, 0.04 mmol of CuBr/PMDETA catalyst system was added under inert conditions, followed by 20 mmol of styrene monomer. The polymerization was conducted at 90 °C for 4 h before termination and purification. After polymerization, the reaction was quenched in an ice bath to stop further growth, followed by standard purification procedures.

For all polymerization reactions, catalyst/ligand combinations of CuCl and Me6TREN were also applicable with the same molar quantities.

### Purification

The products were first purified by vacuum filtration using appropriate solvents, which were IPA for MXene–polymer samples with P4VP as the end block and acetone for the samples having PtBA or PS as the end block, to remove any unreacted monomers and catalyst residues. The filtered products were then subjected to cycles of centrifugation and redispersion in a fresh solvent at least 3 times to ensure the complete removal of any unattached polymer chains and remaining impurities. The final products were dried under vacuum at room temperature.

### Preparation of Linear P4VP Chains for the Comparison Experiment

For stability comparison testing of our surface-grafted MXene-*g*-P4VP and physically adsorbed MXene-P4VP, linear P4VP chains were first fabricated using EBIB as an initiator. Briefly, in 100 mL pressure vessel, 10 mL of EBIB was dispersed in 10 mL IPA, followed by the addition of CuCl, Me6TREN, and 4VP monomer with a relative molar ratio of (EBIB:CuCl:Me6TREN:4VP = 1:1:1:500). After addition, the vessel was resealed and fluxed with inert gas for 1 h. Finally, the polymerization was conducted at 60 °C for a desired time under continuous stirring. The resulting solution was quenched in an ice bath, followed by filtration via an alumina column and multiple washes with IPA and acetone. The final product was collected by vacuum drying in an oven at room temperature.

### Preparation of the Physically Adsorbed MXene-*g*-P4VP Sample for Comparison

Reference MXene-*g*-P4VP samples in which the prefabricated linear P4VP chains are physically adsorbed onto the MXene were prepared via a simple mixing process. Briefly, in a 100 mL round-bottom flask, 10 mg of pristine Ti_3_C_2_T_*x*_ MXene was dispersed in water with short sonication. After dispersion, 20 mg of the linear P4VP chains in 20 mL of IPA was added to the solution, followed by at least 6 h of continuous stirring under an inert gas flux to allow adsorption to the surface. Finally, the resulting product was collected via vacuum filtration.

### Solvent-Resistibility Test of Physically Adsorbed and Surface-Grafted MXene-*g*-P4VP Samples

To compare the durability of the surface to the polymer bonding of different systems, the first 10 mg of the two previously prepared types of MXene-*g*-P4VP samples were dispersed in 10 mL of IPA with short sonication, followed by continuous stirring. After the samples were well dispersed, an excess amount of IPA was added, and then the solution was centrifuged at 6000 rpm for 3 min. Next, the supernatant solution was discarded, and the precipitated flakes were redispersed in IPA. This step was repeated 5 times to simulate harsh environmental stress on MXene–polymer bonding. The final product was dried in a vacuum oven at room temperature.

### Solvent Vapor Annealing of MXene-*g*-P4VP-*b*-PS

MXene-*g*-P4VP-*b*-PS deposited on a Si wafer was subjected to solvent vapor annealing to investigate the self-assembly behavior of the grafted diblock copolymer shells. Samples were prepared by drop-casting dilute suspensions (0.1 mg/mL) of MXene-*g*-P4VP-*b*-PS onto silicon substrates and allowing them to dry under ambient conditions for 24 h. The prepared samples were then placed in a vacuum oven alongside a small container (10 mL) of toluene, a selective solvent for the PS block. The chamber was partially evacuated to promote vapor saturation and maintained at 60 °C for 2 h to facilitate polymer chain mobility and microphase separation. The morphological changes and emergence of self-assembled nanodomains were subsequently characterized by AFM in tapping mode to analyze the thickness changes and surface topography evolution.

### Characterization

TEM analysis was performed using a Hitachi HT7700 operating at an acceleration voltage of 120 kV to examine the morphology and interlayer structure of pristine and functionalized MXene flakes. Samples for TEM were prepared by drop-casting dilute suspensions (0.01 mg/mL) onto carbon-coated copper grids and allowing them to dry under ambient conditions for 24 h.

SEM analysis was conducted on a Hitachi SU8230 and S3400 field emission scanning electron microscope operating at 5 kV to investigate surface morphology and topographical features of the MXene–polymer composites. For SEM analysis, samples were prepared by depositing the material onto silicon wafers and sputter-coating with a 3 nm layer of platinum to enhance conductivity and prevent charging effects. Energy-dispersive X-ray spectroscopy (EDX) at 10 kV was performed alongside SEM measurements to analyze the elemental composition and distribution across the functionalized MXene surfaces, with particular attention to bromine content following the surface modification step.

WAXS analysis was done using a Rigaku Smartlab XE in a parallel beam geometry mode. The X-ray was equipped with a HyPix-3000 2D detector utilizing X-ray radiation from a Cu anode (wavelength 1.54 Å). The MXene sample was drop-cast onto a Si wafer and placed on a zero-background holder; alignment was performed for each sample measurement. The scanning speed was maintained at 1 °/min, and the tilt angle was fixed at 1°, covering a 2θ range from 3 to 40°.

XPS measurements were performed using a Thermo Scientific K-Alpha spectrometer equipped with a monochromatic Al Kα X-ray source (1486.6 eV). Samples were prepared by drop-casting dispersions of pristine MXene, brominated MXene (MXene-Br), and various MXene–polymer composites onto silicon wafers, followed by drying under vacuum at room temperature for 24 h. Survey scans were conducted with a pass energy of 200 eV, while high-resolution scans for Ti 2p, C 1s, O 1s, N 1s, and Br 3d regions were performed with a pass energy of 50 eV. All spectra were calibrated using the C 1s peak at 284.8 eV as a reference.

FT-IR analysis was conducted using a Bruker Vertex 70 spectrometer equipped with an attenuated total reflectance (ATR) accessory. Samples of pristine MXene, brominated MXene, and various MXene–polymer composites were thoroughly dried under vacuum for 24 h to remove any residual solvents. Measurements were performed in the wavenumber range of 4000–1200 cm^–1^ averaging 128 scans per sample to improve signal-to-noise ratio. For polymer-grafted MXene samples, where MXene’s strong infrared absorption could potentially obscure polymer signals, sufficient polymer content was ensured through extended polymerization times to obtain clear polymer characteristic peaks. Background spectra were collected before each measurement and automatically subtracted from the sample spectra. When necessary, baseline correction was applied using OPUS software to improve peak visibility without distorting spectral features.

GPC was conducted using a Shimadzu LC-20 HPLC to determine the molecular weight and polydispersity index of polymer chains. As direct GPC analysis of MXene-grafted polymers was not feasible, a cleavage step was performed to release the polymer chains. The MXene–polymer composite was stirred in a 1 M HCl solution (methanol/water 1:1 v/v) at room temperature for 12 h and followed by filtration and drying to retrieve cleaved polymer chains. After the process, the dried polymer was dissolved in DMF and filtered through a 0.22 μm PTFE membrane, then finally analyzed by GPC with polystyrene standards for calibration.

Surface morphology and film thickness were analyzed using a Bruker Dimension Icon atomic force microscope. Samples were prepared by applying the blade-coating method to diluted samples on a Si substrate cleaned with standard piranha solutions. Measurements were conducted in Standard Tapping mode in air with standard AFM tips, using 512 samples per line and a scanning rate of 0.5 and 1.5 Hz. Scans were performed over 1.5 × 1.5 μm and 0.75 × 0.75 μm and other different-sized regions following standard procedures. Roughness measurements were conducted over multiple 75 nm × 75 nm areas chosen within the boundaries of individual flakes, accurately characterizing the polymer surface without edge effects. Postprocessing, including image flattening, height measurements, cross-sectional height analysis, and microroughness calculations, was performed using NanoScope Analysis 2.0 software.

Raman spectra measurement was conducted using WiTEC alpha300 R confocal Raman microscope to analyze the vibrational modes and oxidation state of the prepared samples. Prior to measurement, the samples were prepared by depositing the target material onto a glass substrate covered with aluminum foil to minimize background noise and enhance signal clarity. A drop-casting method was employed for deposition, followed by controlled drying to achieve uniform distribution. The microscope was operated with a 532 nm excitation laser, and spectral acquisition was performed using a high-sensitivity CCD detector with an appropriate grating selection to resolve characteristic peaks with high precision.

## Supplementary Material



## Notes

Additional experimental data, including TEM, XPS, FTIR, GPC, TGA, and AFM images of pristine, brominated, and polymer-grafted MXene not included in main figures; AFM height and roughness profiles; solvent vapor annealing AFM analysis; and a table of polymer brush layer thickness and grafting density with previously reported data from literature for comparison (PDF)
