# Supplementary material for: Precision Grafting-From of Diblock Copolymer Brushes on MXene Nanosheets
Source: Chem Mater. 2025 Oct 13;37(20):8238–51. doi: 10.1021/acs.chemmater.5c01572 (PMC12573581; doi:10.1021/acs.chemmater.5c01572)
Supplement: Supplementary file 1 [file cm5c01572_si_001.pdf]

Supporting Information

Precision Grafting-From of Diblock Copolymer Brushes on MXene Nanosheets

Jinyoung Choi[1]*, Mykhailo Yelipashev*[1]*, Valeriia Poliukhova*[1]*, James FitzPatrick*[2]*, Yury Gogotsi*[2]*, Zhiqun Lin*[3]*, Vladimir V. Tsukruk*[1]

[1]School of Materials Science and Engineering, Georgia Institute of Technology, Atlanta, Georgia 30332, United States
[2]A. J. Drexel Nanomaterials Institute and Department of Materials Science and Engineering, Drexel University, Philadelphia, Pennsylvania 19104, United States
[3]Department of Chemical and Biomolecular Engineering, National University of Singapore, Singapore, 117585 Singapore

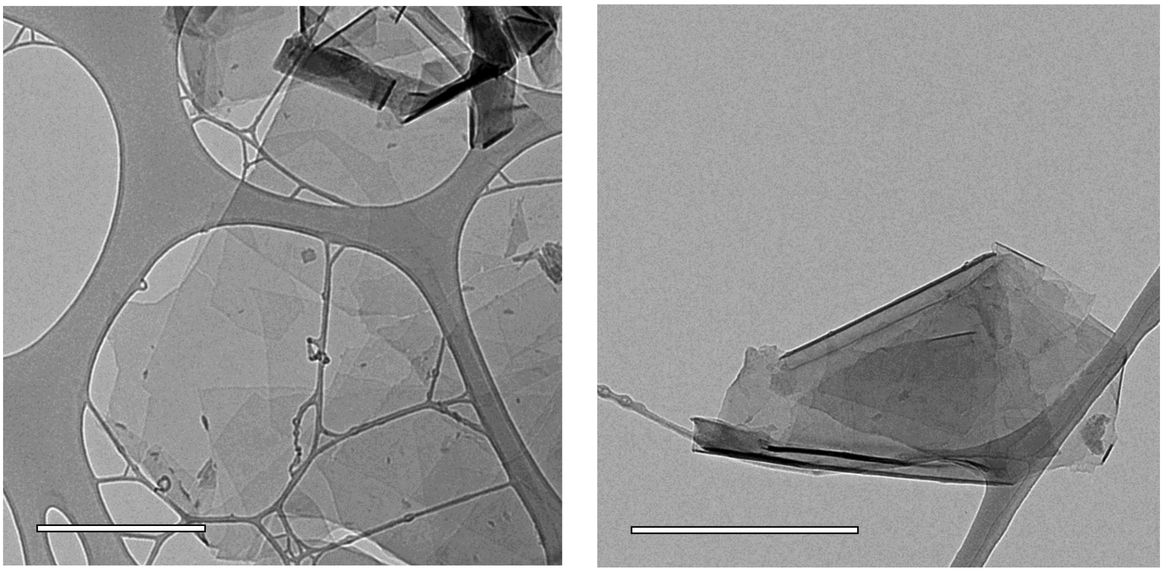

Figure S1. TEM image of pristine $Ti_3C_2T_x$ MXene flakes with flake sizes ranging from 500 nm to several micrometers.

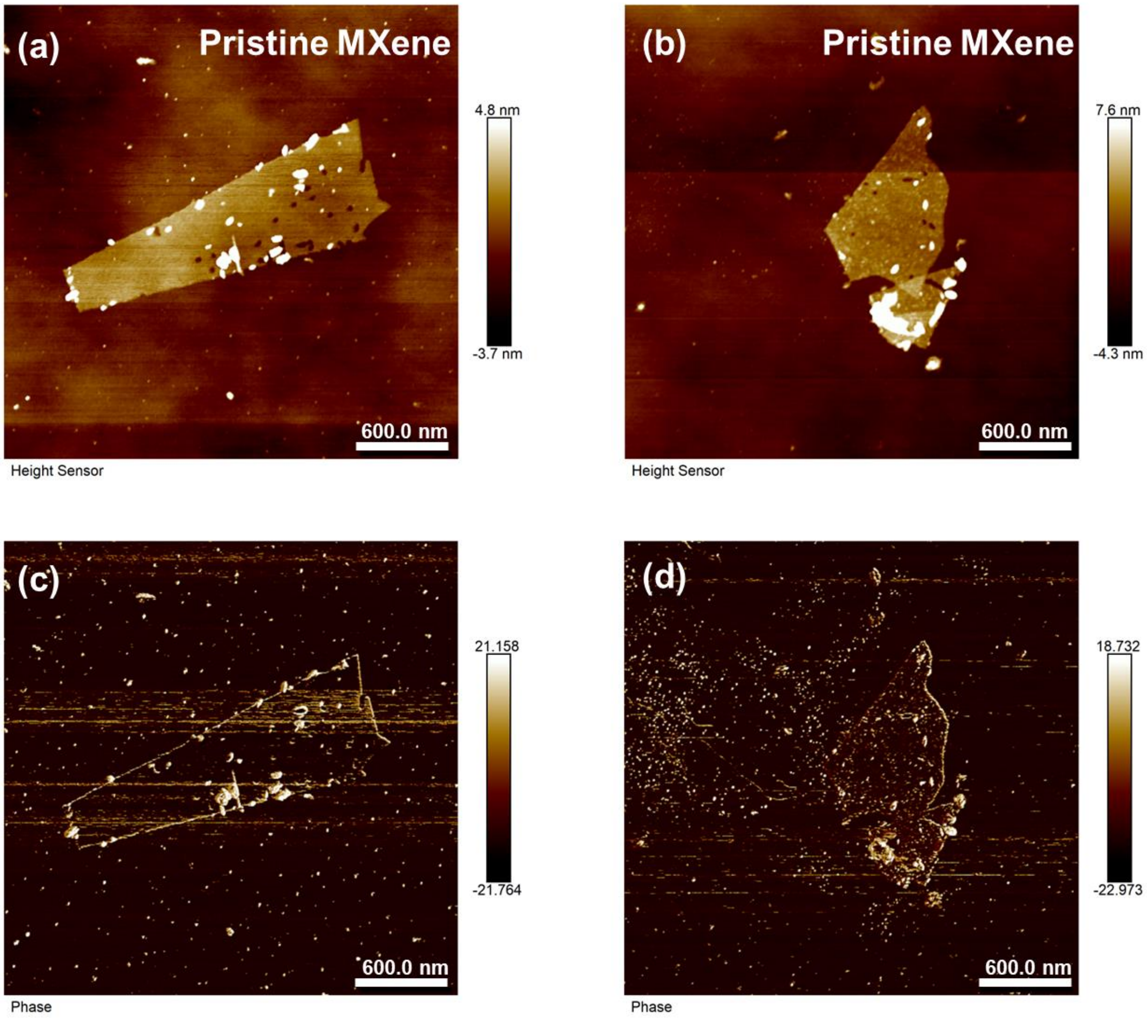

Figure S2. AFM images of pristine single MXene flakes with a lateral length of approximately 2 μm. (a), (b) show height sensor images, and the lower figures (c), (d) are respective phase images.

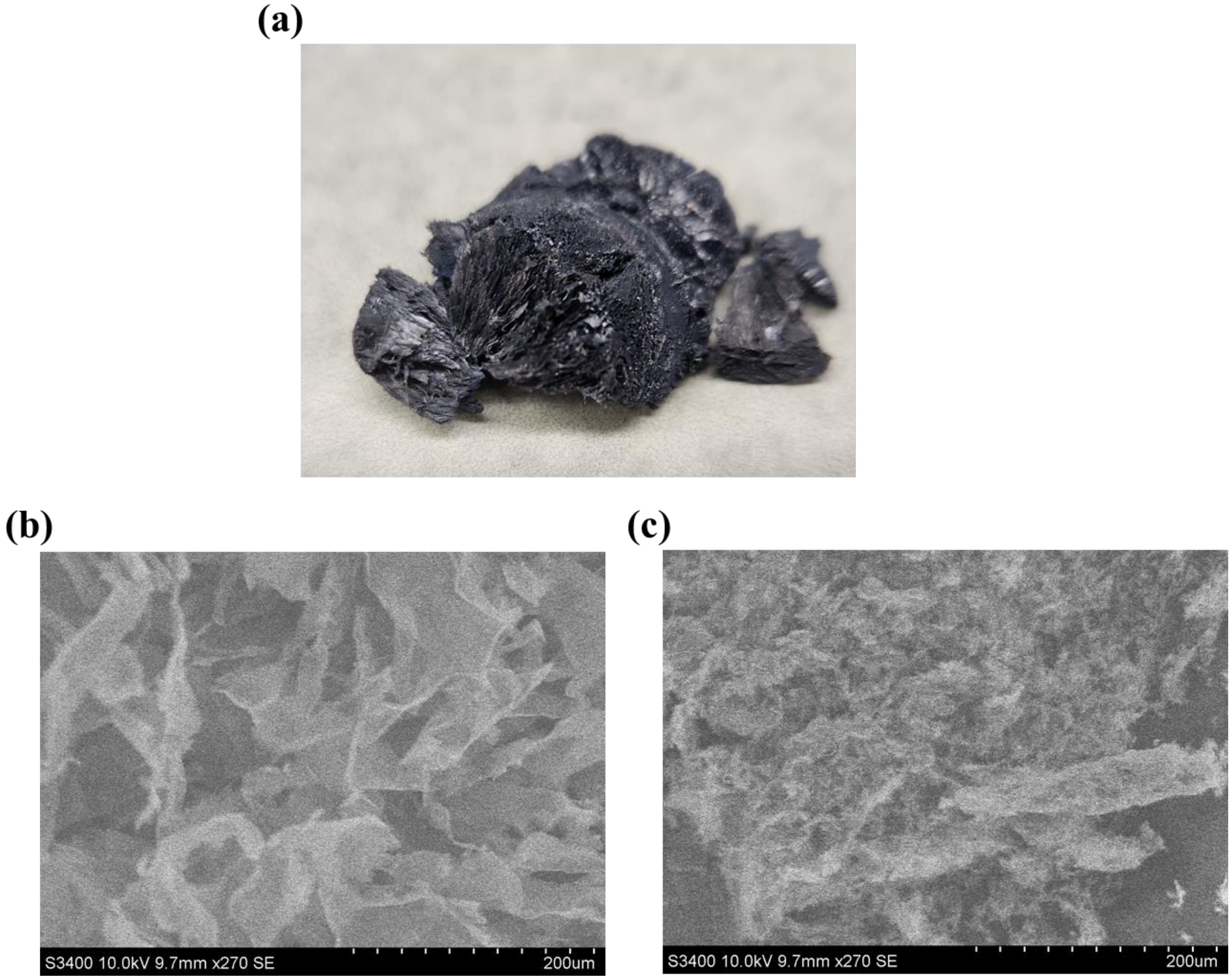

Figure S3. Morphology of MXene after liquid N_2 rapid cooling and freeze-drying to maximize surface exposure. (a) digital photograph of pristine MXene retrieved after liquid N_2 rapid cooling process followed by freeze-drying and SEM image of freeze-dried MXene (b) without and (c) with rapid cooling. Highly porous, sponge-like appearance can be observed for MXene treated with liquid N_2.

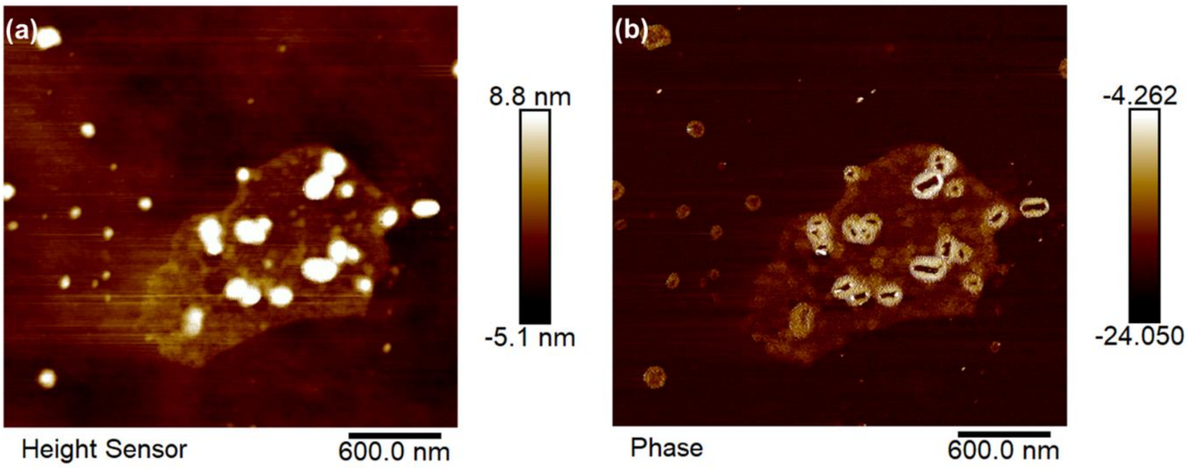

Figure S4. AFM (a) height and (b) phase image of brominated MXene flakes, with preserved 2D morphology after the bromination step. Bright white spots are external impurities attached from outside.

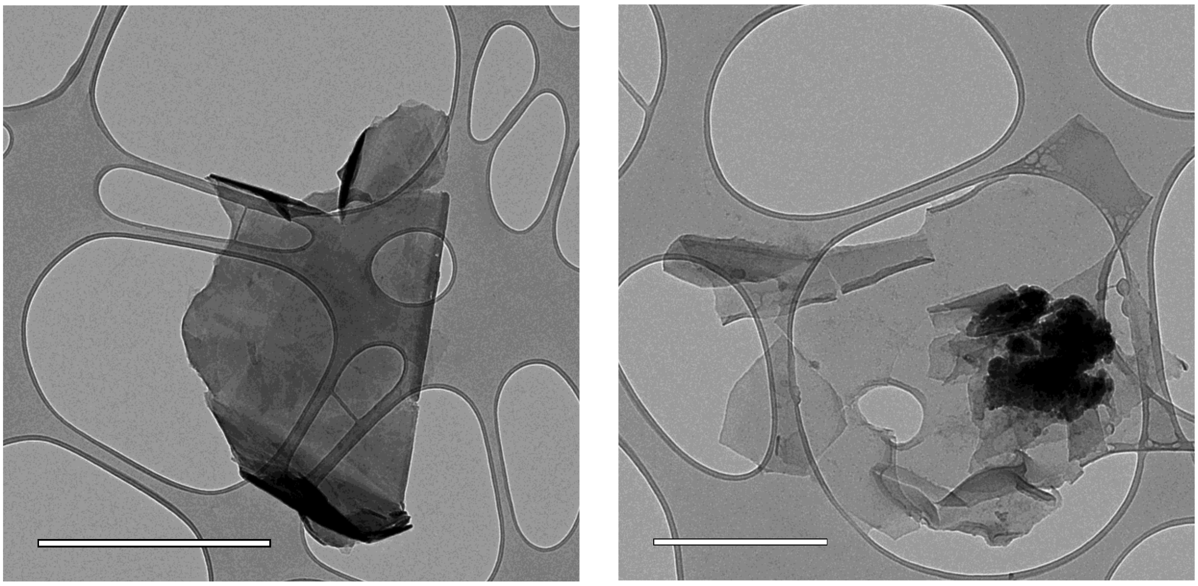

Figure S5. TEM images of brominated MXene flakes, showing preserved 2D morphology of the flakes after bromination.

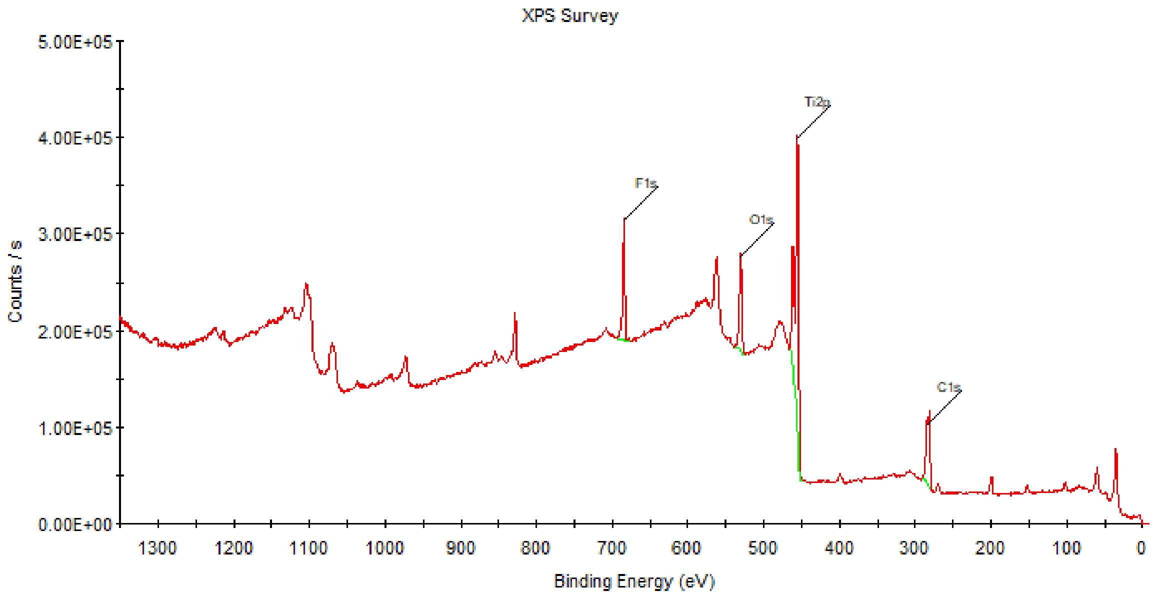

Figure S6. XPS Survey spectra of pristine MXene, with major signals from Ti 2p, O 1s, and C 1s are marked.

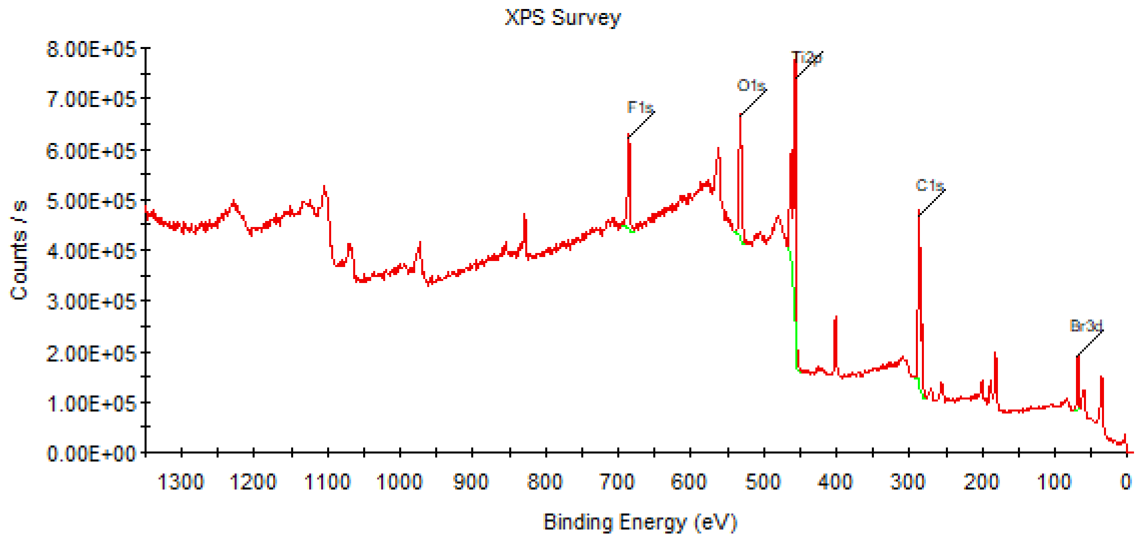

Figure S7. XPS survey spectra of brominated MXene, with major signals from Ti 2p, O 1s, C 1s, and Br 3d are marked. A clear, sharp Br 3d peak originating from the attached 2-BIBB can be observed.

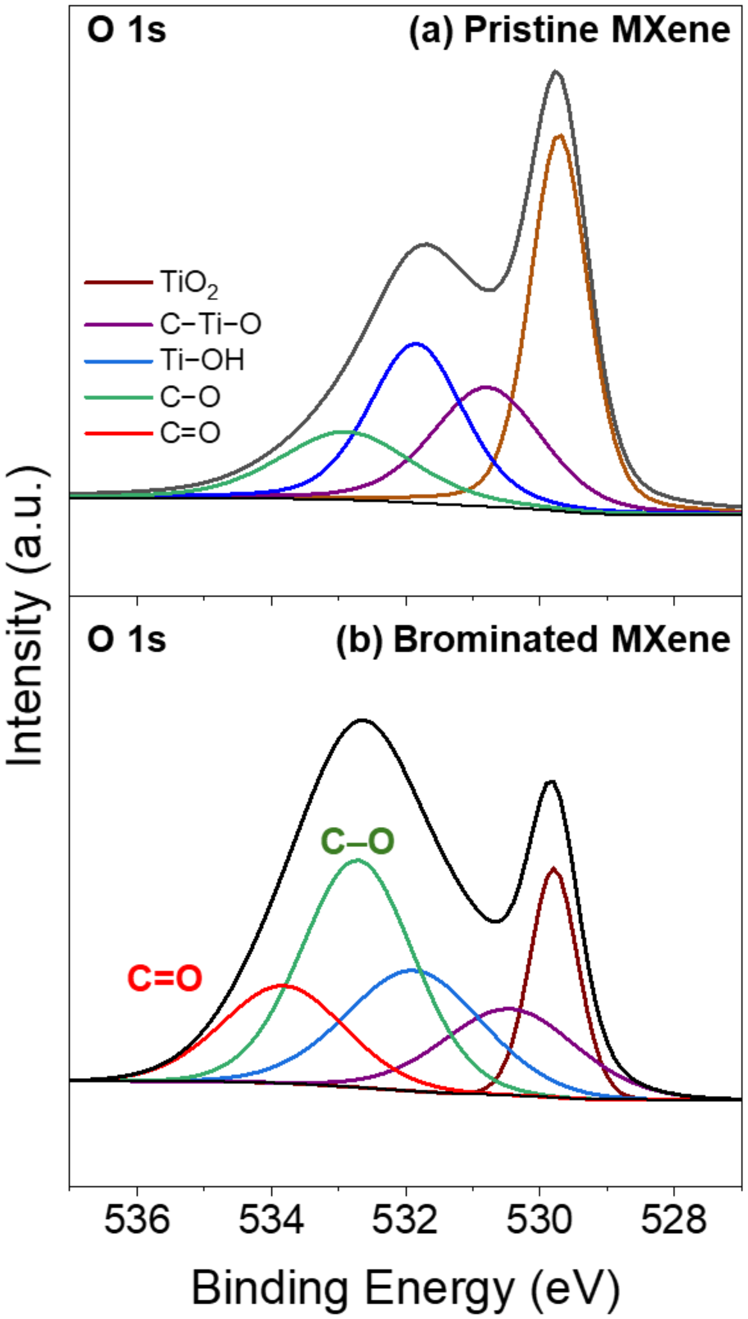

Figure S8. O 1s XPS spectra of (a) Pristine and (b) Brominated MXene, where the legend for convoluted peaks is shown on the upper left side. Compared to pristine MXene, the brominated MXene shows a decrease in -OH peak, a huge increase in C–O peak, and the appearance of a significant C=O peak, all originating from the attached 2-BIBB initiator replacing –OH sites.

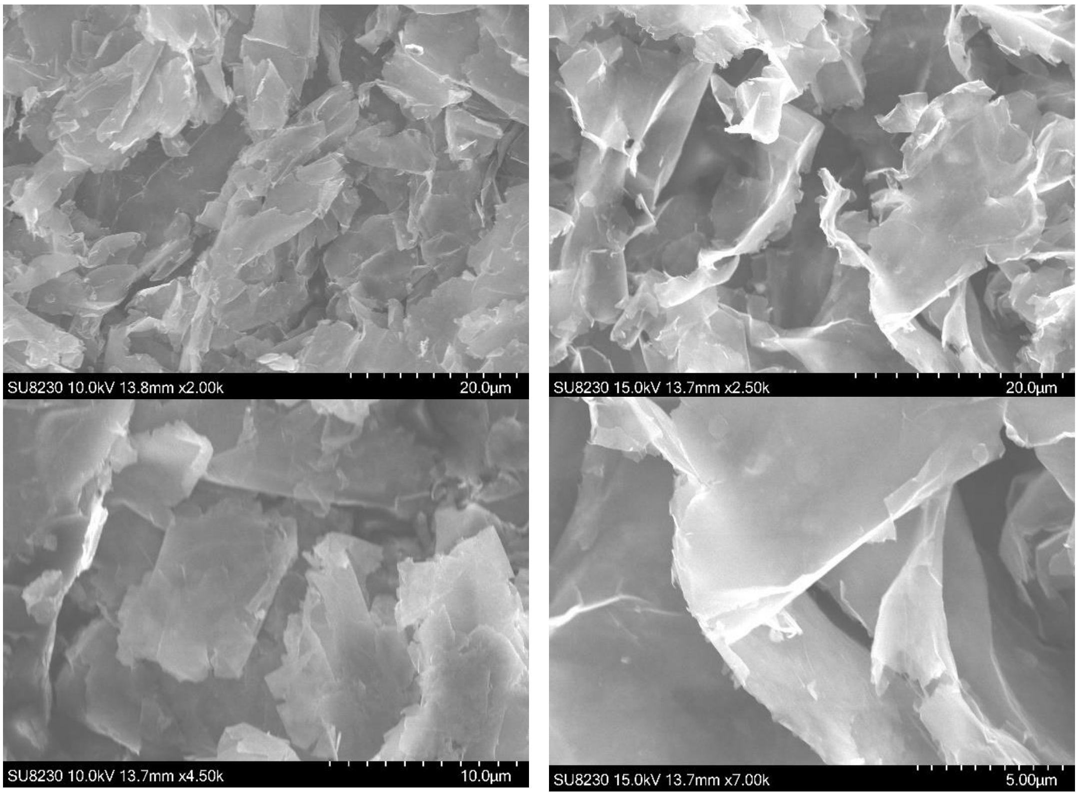

Figure S9. HRSEM image of brominated MXene flakes with different magnification, displaying preserved 2D structure after the bromination step.

(a)

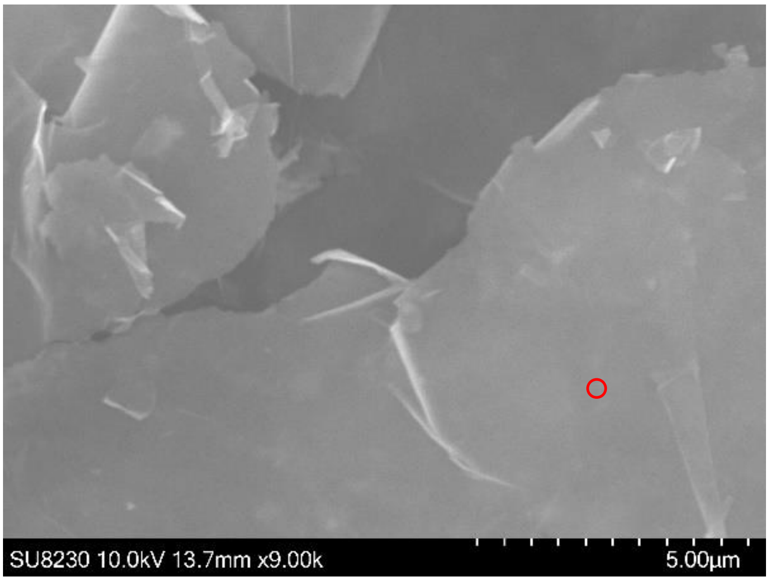

(b)

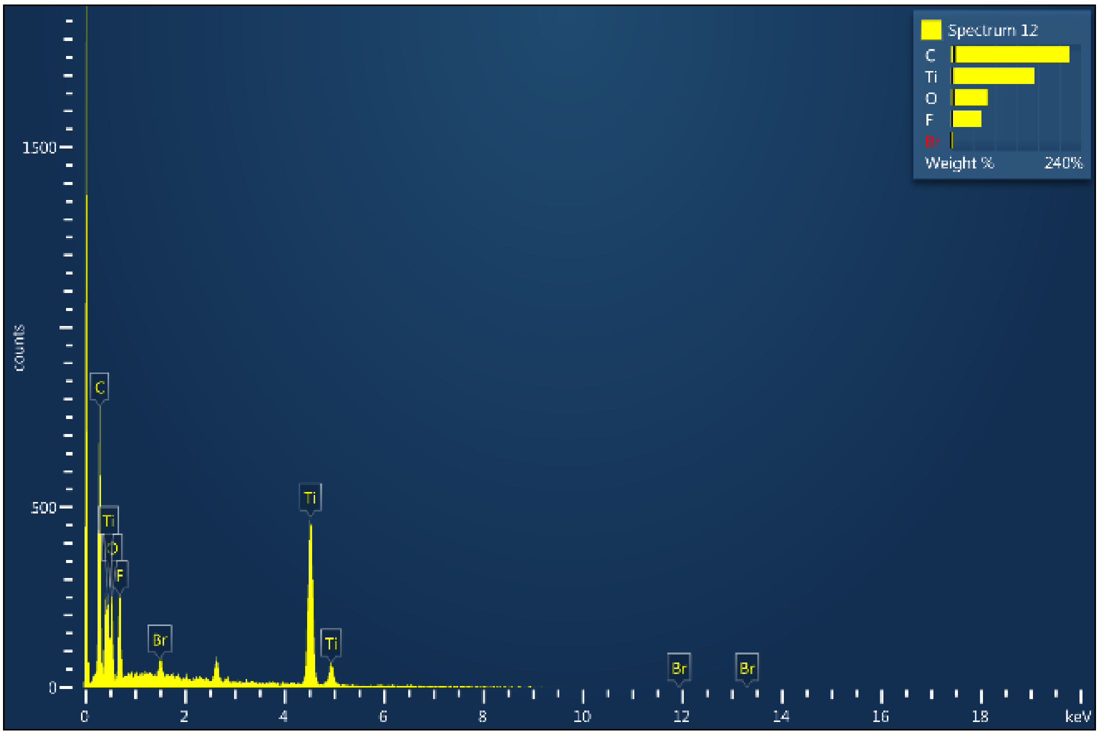

Figure S10. (a) HR SEM image and (b) spot EDS plot of brominated MXene flakes. The position of the spot is marked as a red dot in (a). A certain presence of the Br element originated from the 2-BIBB can be observed.

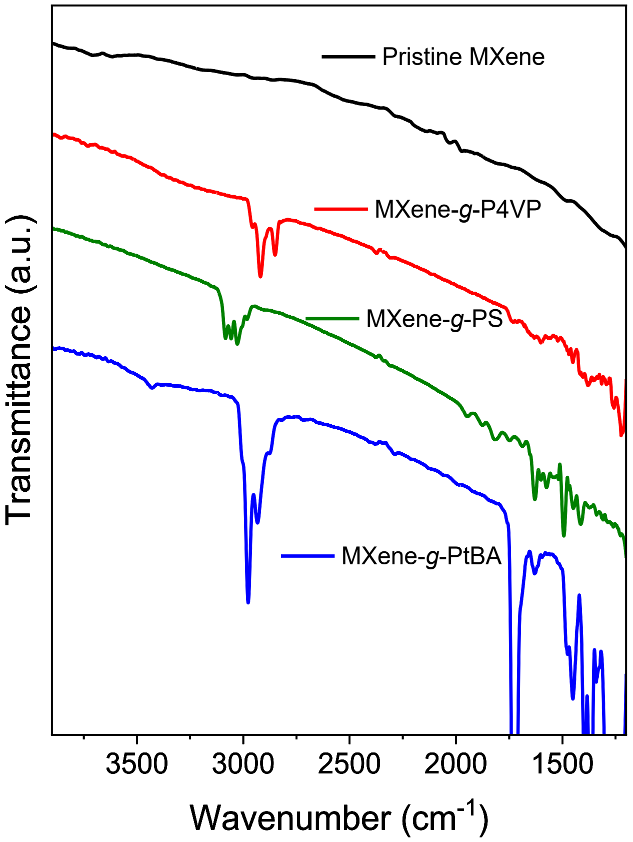

Figure S11. FTIR spectra of pristine and MXene-polymer samples without background deduction. MXene-Polymer samples resemble distinct polymer-derived peaks even before signal processing, particularly for the 2800 cm^{-1} to 3200 cm^{-1} regions corresponding to C-H vibrations.

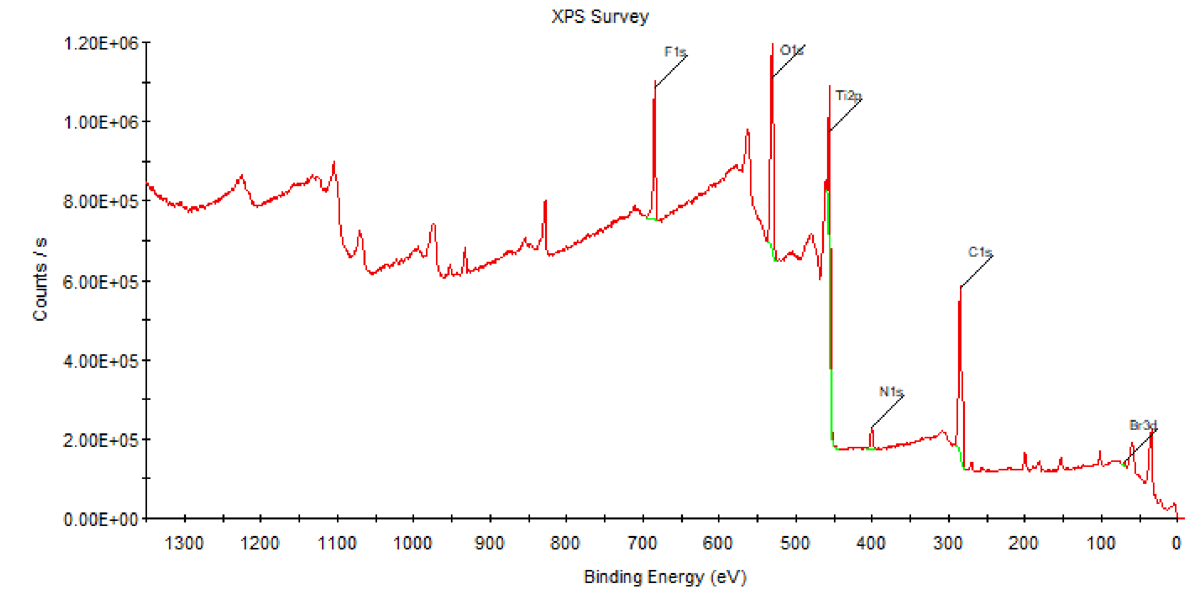

Figure S12. XPS Survey spectra of MXene-*g*-P4VP with major peaks labelled. N 1s peak and the maintained presence of Br 3d peak can be observed.

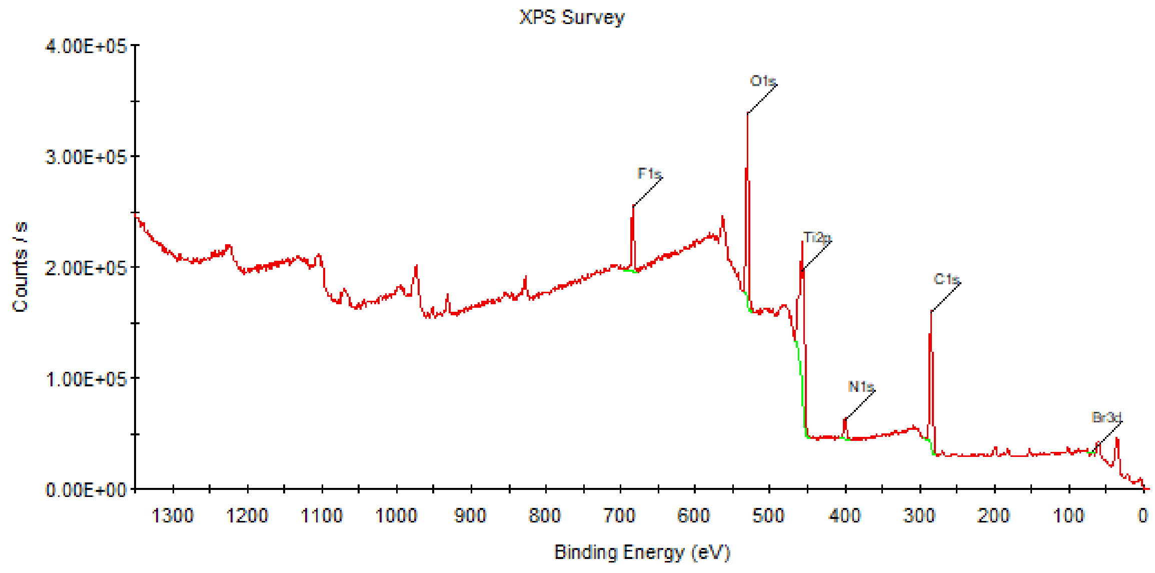

Figure S13. XPS Survey spectra of MXene-*g*-P4VP-*b*-PtBA. The maintained presence of the Br 3d peak can be observed.

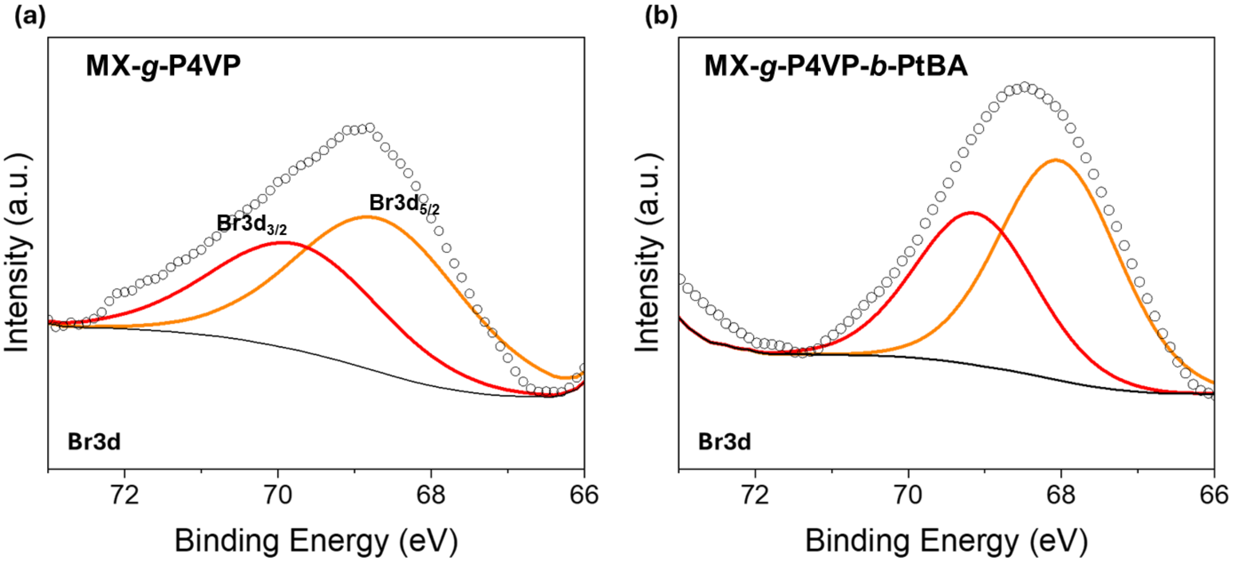

Figure S14. Br 3d XPS spectra of (a) MXene-*g*-P4VP and (b) MXene-*g*-P4VP-*b*-PtBA samples synthesized using CuBr as ATRP catalyst, with doublet deconvolution. Both samples show maintained presence of Br, indicating the polymer brushes still have the bromine attached to their end, which can continue to act as initiator sited for further polymer block growth.

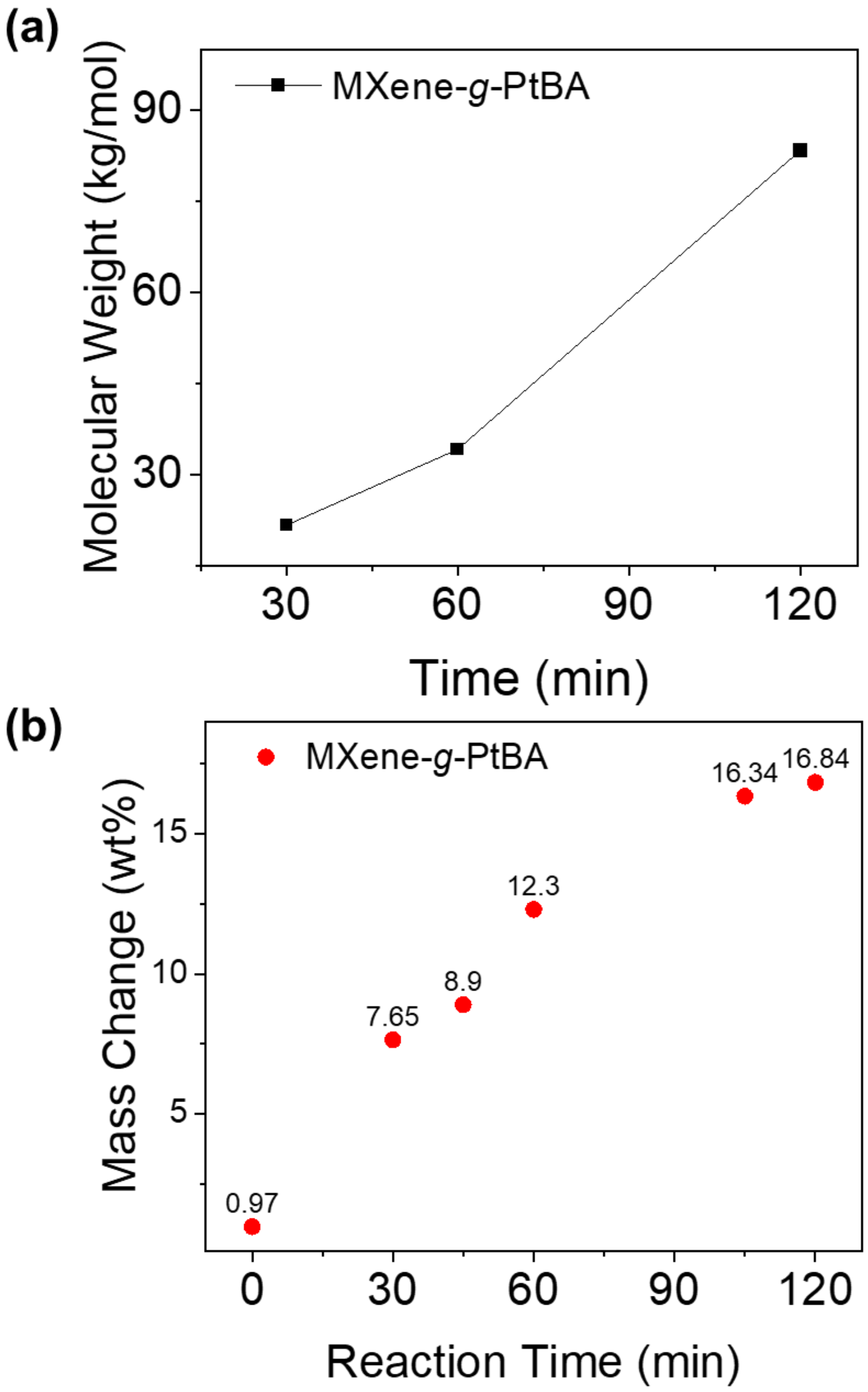

Figure S15. Length characterizations of PtBA brushes grown on the MXene surface with different reaction times. (a) Molecular weight of single PtBA brushes cleaved from the surface analyzed by GPC, (b) relative mass change of MXene-*g*-PtBA samples observed by TGA. The expected increase in length and mass ratio of polymer chains over time can be observed.

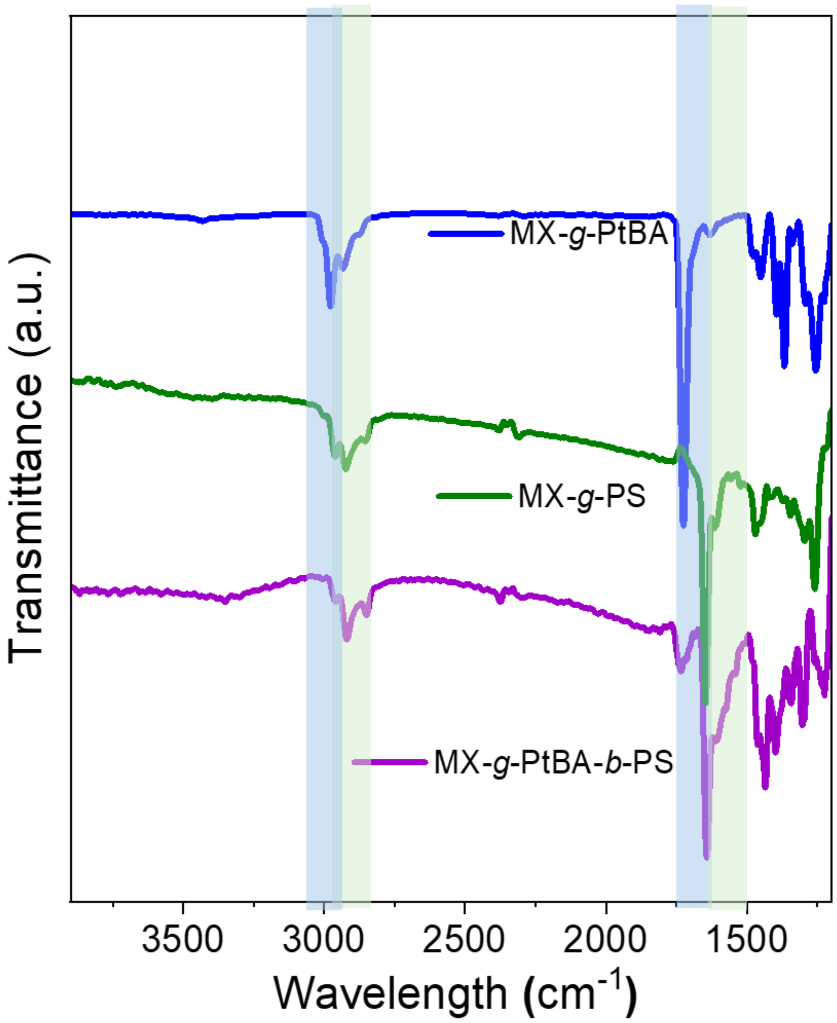

Figure S16. FT-IR spectra comparison of MXene-*g*-PtBA / MXene-*g*-PS with single block polymer brushes, and MXene-*g*-PtBA-*b*-PS with diblock copolymer after background deduction. Inheritance of characteristic peak positions from each block to the diblock system can be observed.

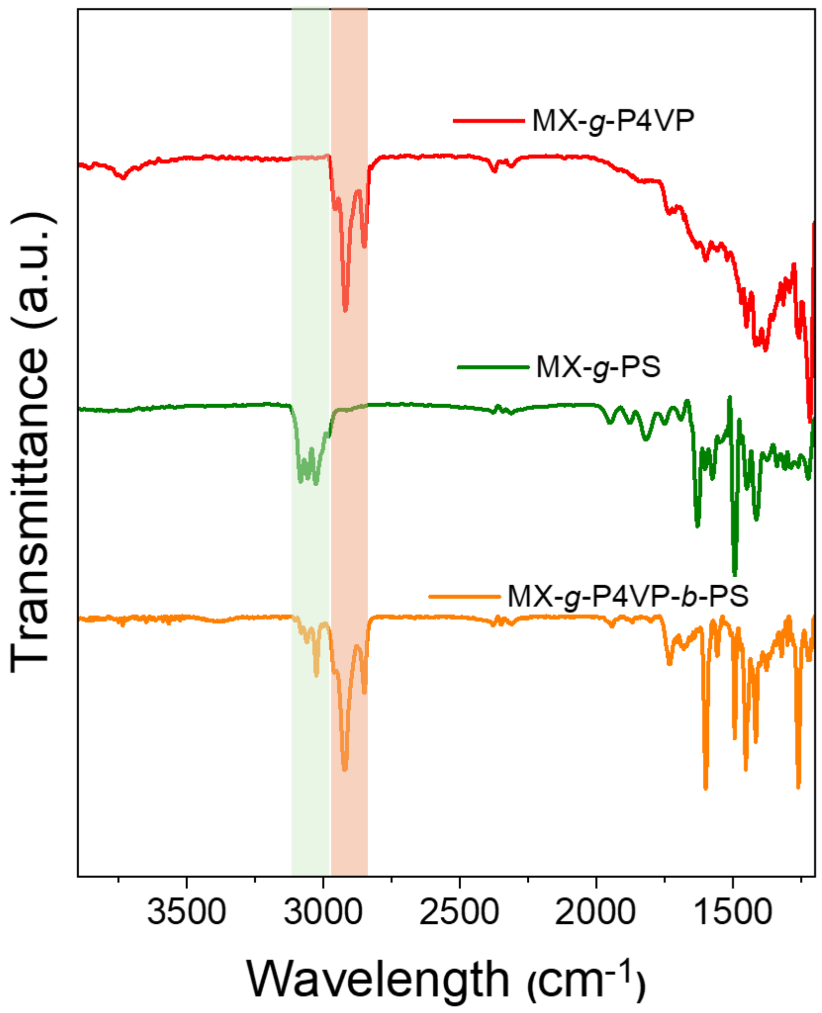

Figure S17. FT-IR spectra comparison of MXene-*g*-P4VP / MXene-*g*-PS with single block polymer brushes, and MXene-*g*-P4VP-*b*-PS with diblock copolymer after background deduction. Inheritance of characteristic peak positions from each block to the diblock system can be observed.

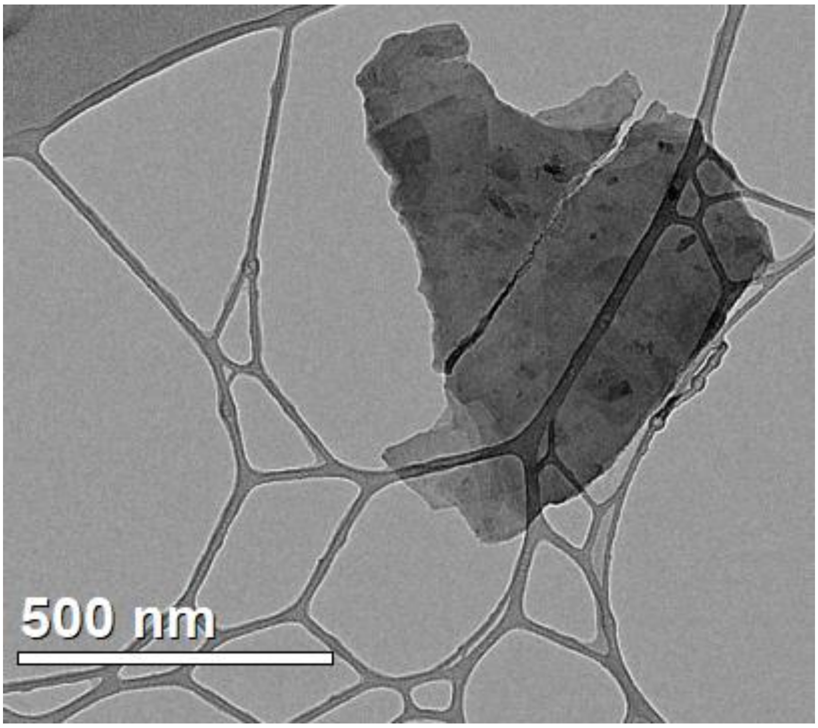

Figure S18. TEM image of a MXene-*g*-P4VP-*b*-PtBA sample, showing that no distinct differences from the pristine flake are observed due to the low contrast of polymer shells compared to MXene.

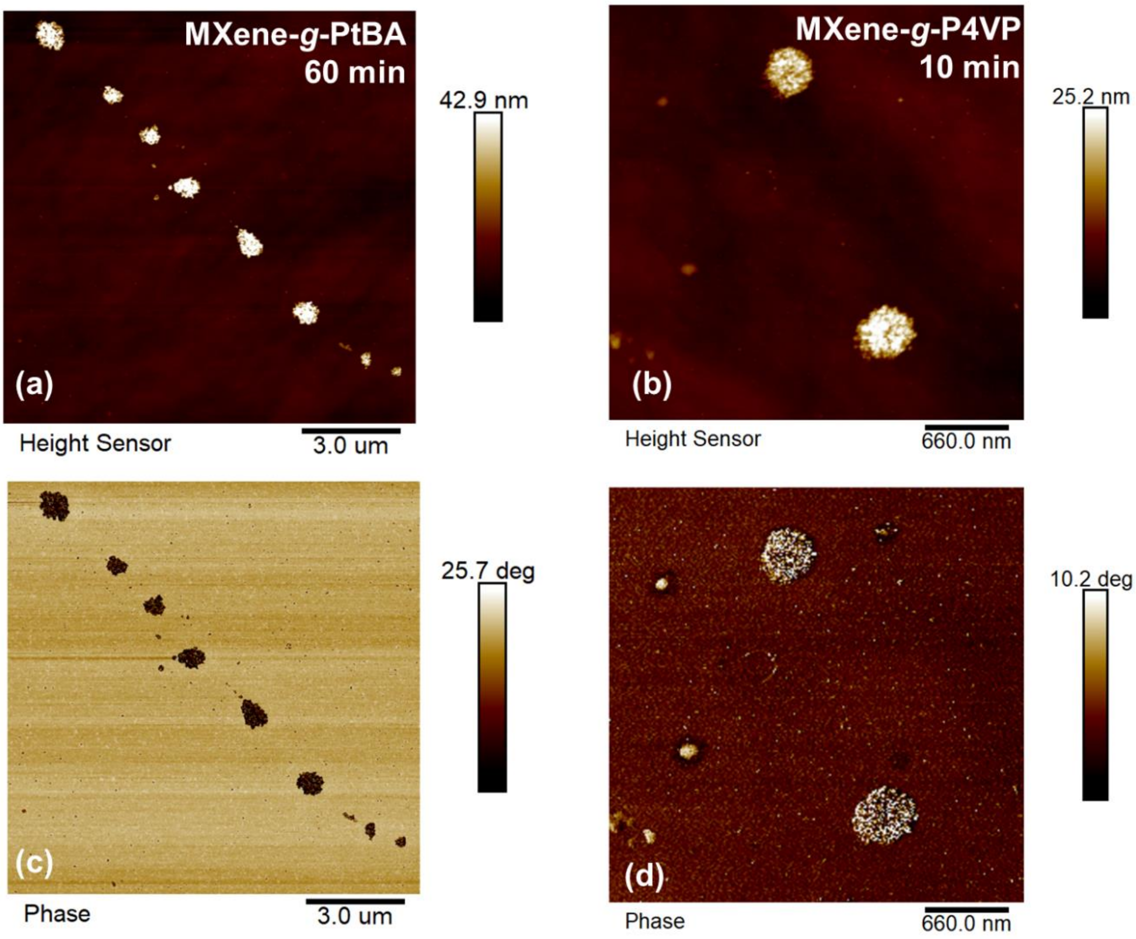

Figure S19. AFM image of (a), (c) MXene-*g*-PtBA and (b), (d)MXene-*g*-P4VP samples obtained by shear deposition with multiple flakes in one view, displaying uniformity over different flakes of our sample. Height sensor images are on the upper side, and corresponding phased images are displayed on the lower side.

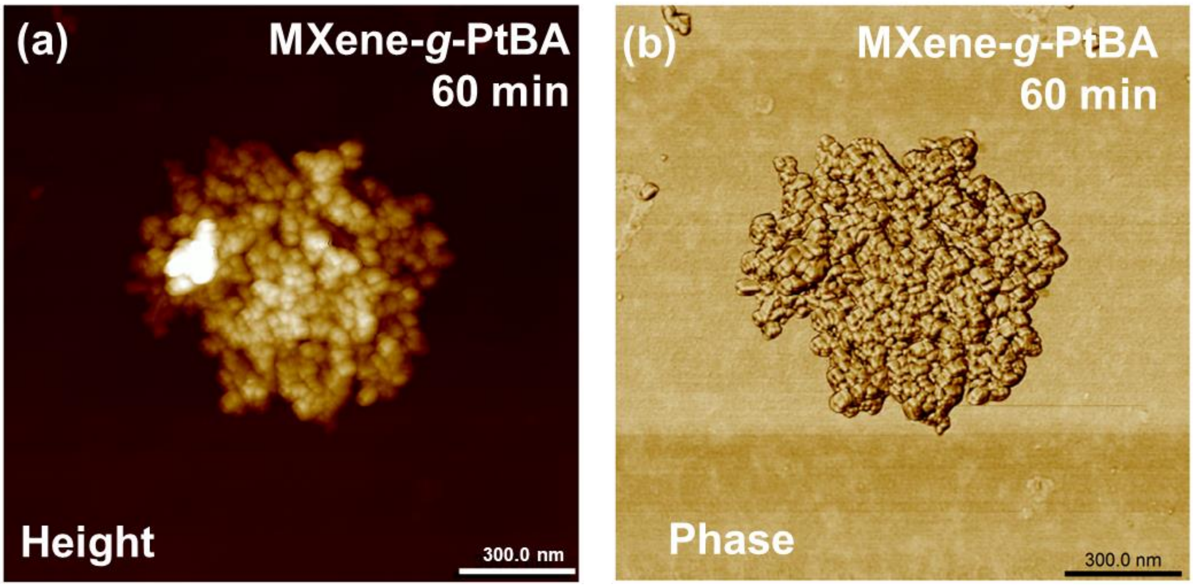

Figure S20. AFM (a) height and (b) phase images of MXene-*g*-PtBA samples with a reaction time of 60 minutes.

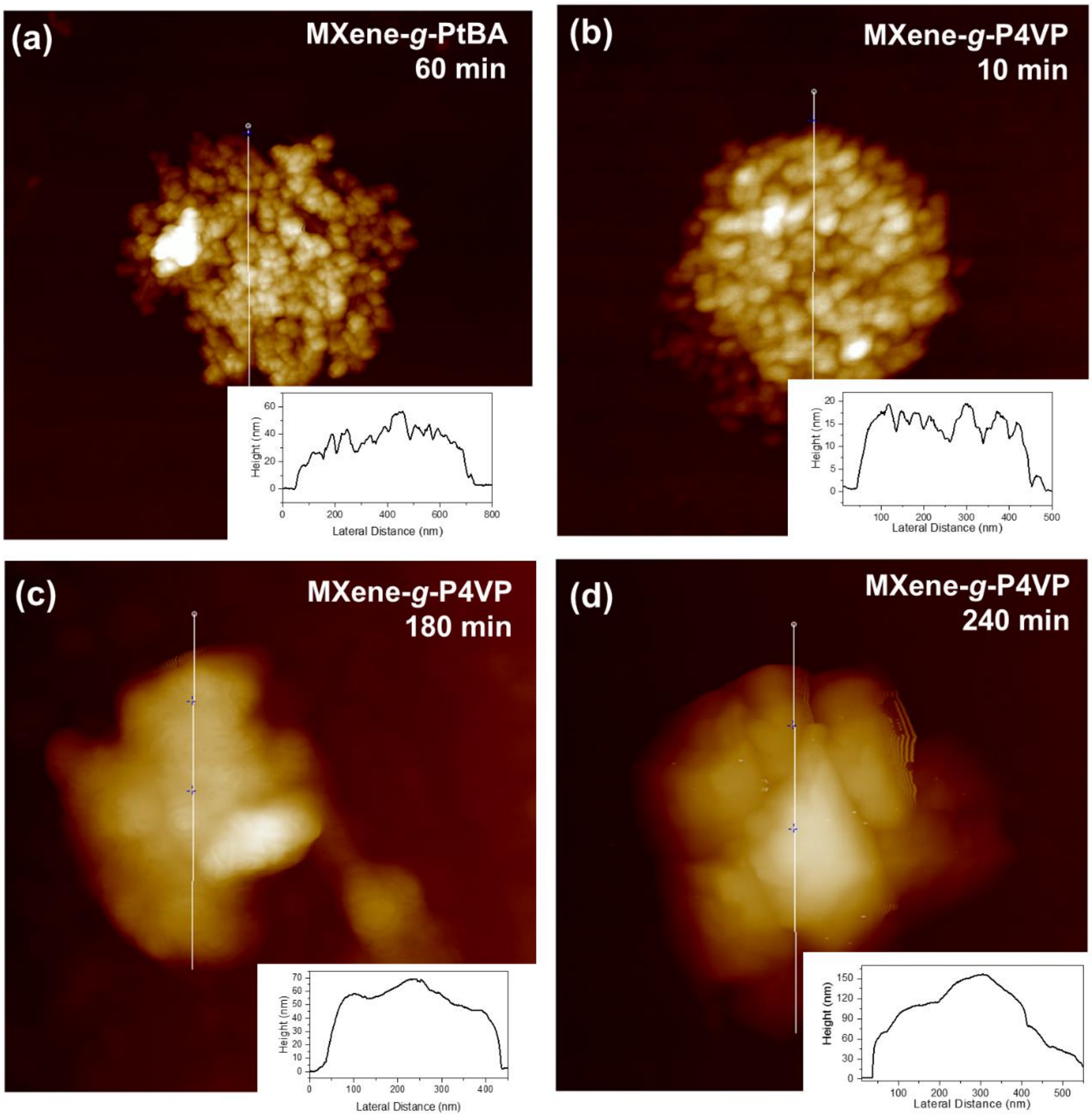

Figure S21. The sectional height profile of MXene-Polymer samples with different chain lengths obtained through AFM. (a) MXene-*g*-PtBA reacted for 60 minutes, (b) MXene-*g*-P4VP for 10 minutes, (c) 180 minutes, and (d) 240 minutes. Insets show a depth profile plot along the slice line drawn on the image. As the molecular weight and length of the polymer chain increase with reaction time, a much smoother surface due to the packing of brushes can be observed.

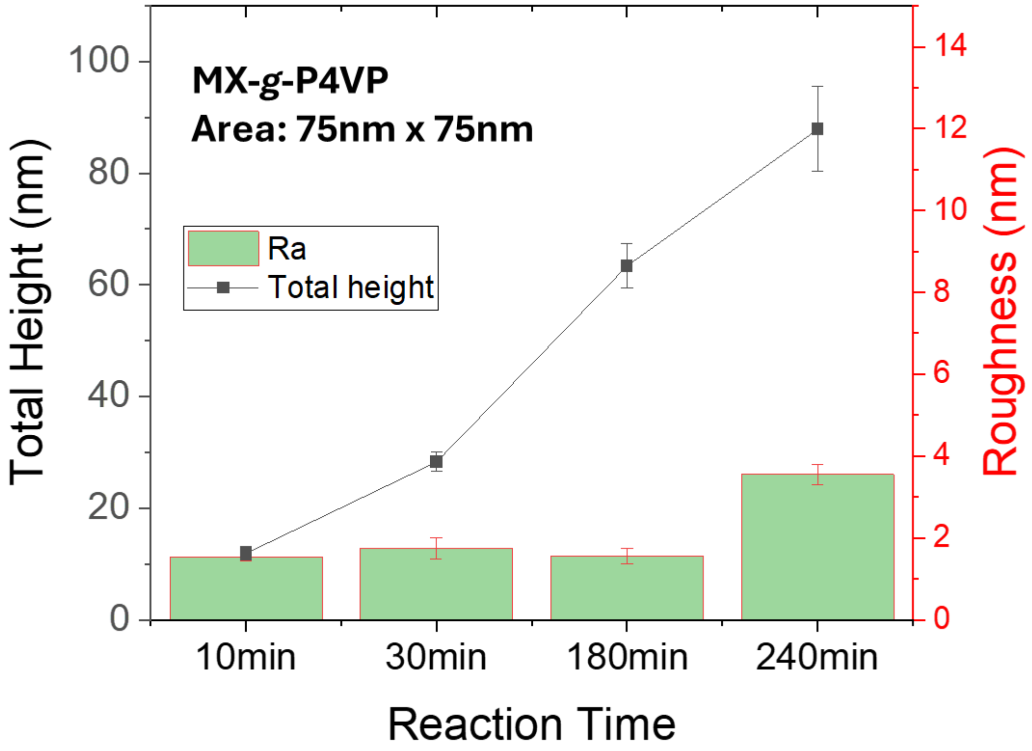

Figure S22. Total height of MXene-polymer composite measured by AFM and roughness plot of MXene-*g*-P4VP samples with different reaction times ranging from 10 to 240 minutes, showing maintained roughness value kept to below ~10 % of the samples' total heights. The measurement area for roughness calculation is also shown.

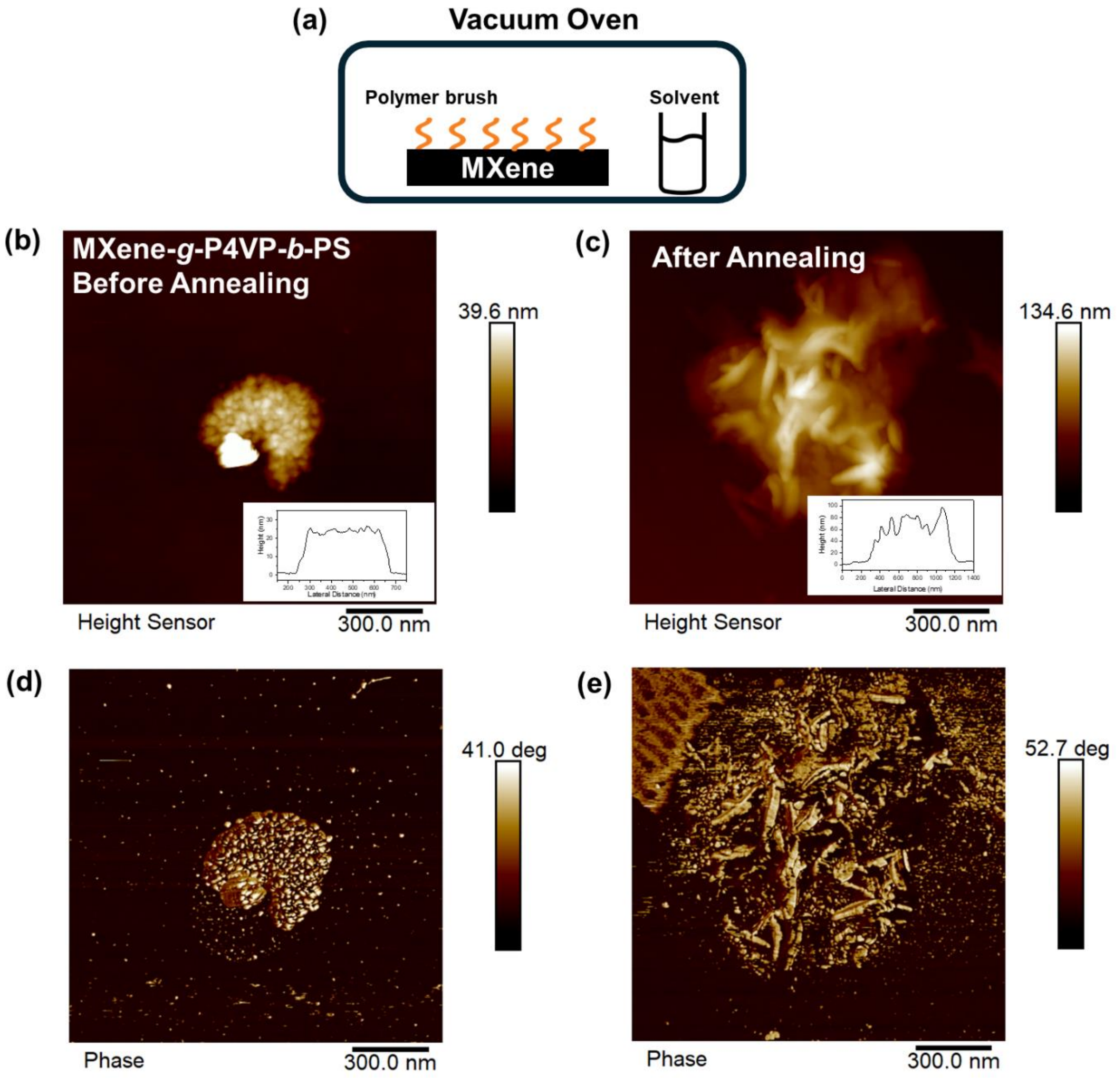

Figure S23. Effect of toluene solvent vapor annealing on MXene-*g*-P4VP-*b*-PS samples characterized by AFM. The schematic setup of the solvent vapor annealing process is also displayed on (a). Height sensor images for (b) before annealing and (c) after annealing are on the upper side, and corresponding phased images (d), (e) are displayed on the lower side. A certain morphological shift to dendrite-like shape formation, likely originating from swelling of the PS block, can be observed.

Table S1. Thickness of polymer brush layer measured by AFM, with calculated grafting density for samples with GPC measured molecular weight. Values from other research with similar molecular weights of polymer brushes with comparable grafting densities are also provided for reference.[1,2,3]

Source	Sample Name or Description	GPC measured molecular weight (g/mol)	AFM measured thickness (nm) (Single Side)	Calculated grafting density (chains / nm^2)
This Paper	**MX-*g*-P4VP 30m**	45.6K	12.7 ± 0.83	0.192
	**MX-*g*-P4VP 3hr**	89.3K	30.2 ± 3.0	0.181
	**MX-*g*-P4VP 4hr**	137.0K	42.5 ± 3.8	0.239
	**MX-*g*-PtBA 1hr**	34.2K	14.3 ± 1.6	0.252
	**MX-*g*-PtBA 2hr**	83.4K	27.3 ± 2.9	0.197
	**MX-*g*-P4VP-*b*-PtBA**	191.0K	77.2 ± 3.7	0.243
	**MX-*g*-P4VP-*b*-PS**	84.0K	25.2 ± 2.2	0.208
	**MX-*g*-PtBA-*b*-PS**	129.3K	45.1 ± 1.8	0.210
**(1)**	**P(S-r-MMA) on silicon**	19.9K	10.9	0.37
		69.0K	19.0	0.19
**(2)**	**PtBA on silica nanoparticles**	37.1K	39.3 ± 0.2	0.32
		83.2K	57.2 ± 0.3	0.32
**(3)**	**PS on epoxy-modified silicon**	11.9K	2.3	0.12 – 0.21
		45.8K	8.0	0.11

References

[1] Sparnacci, K.; Antonioli, D.; Gianotti, V.; Laus, M.; Ferrarese Lupi, F.; Giammaria, T. J.; Seguini, G.; Perego, M. Ultrathin Random Copolymer-Grafted Layers for Block Copolymer Self-Assembly. *ACS Appl Mater Interfaces* **2015**, *7* (20), 10944–10951. https://doi.org/10.1021/ACSAMI.5B02201

[2] Li, T.-H.; Yadav, V.; Conrad, J. C.; Robertson, M. L. Effect of Dispersity on the Conformation of Spherical Polymer Brushes. **2021**, *20*, 23. https://doi.org/10.1021/acsmacrolett.0c00898.

[3] Luzinov, I.; Tsukruk, V. V. Ultrathin Triblock Copolymer Films on Tailored Polymer Brushes. *Macromolecules* **2002**, *35* (15), 5963–5973. https://doi.org/10.1021/MA0205818/
